# BMP2-Binding Caffeoylquinic Acids from *Periploca forrestii* Promote Osteoblast Differentiation via Smad Signaling Activation

**DOI:** 10.3390/biology15161385

**Published:** 2026-08-13

**Authors:** Minghong Dong, Xinyue Wang, Xiongwei Liu, Tingting Feng, Chang Liu, Ying Zhou

**Affiliations:** 1 School of Pharmacy, Guizhou University of Traditional Chinese Medicine, Guiyang 550025, China; dmh99102@163.com (M.D.); wwwwxy996@163.com (X.W.); ashevy0819@163.com (X.L.); fengtingting040@gzy.edu.cn (T.F.); 2 Guizhou Key Laboratory of Modern Traditional Chinese Medicine Creation, Guiyang 550025, China

**Keywords:** BMP2-Smads signaling, SPR, *Periploca forrestii*, isochlorogenic acid B, osteoblast differentiation

## Abstract

The BMP2-Smad signaling pathway serves as a central regulator of osteoblast differentiation and bone formation, rendering it a promising target for the discovery of osteogenic agents from natural sources. Nonetheless, direct BMP2-binding ligands derived from complex herbal extracts remain poorly characterized. In the present study, surface plasmon resonance (SPR)-based target fishing against BMP2, in conjunction with UPLC-Q-TOF-MS identification, was employed to screen for bioactive ligands from *Periploca forrestii*, a traditional Miao medicinal plant used for bone-related conditions. Six caffeoylquinic acid derivatives, namely neochlorogenic acid (NCA), 3-*O*-caffeoyl-4-*O*-sinapoylquinic acid, chlorogenic acid (CA), cryptochlorogenic acid (CCA), isochlorogenic acid B (IB), and isochlorogenic acid C (IC), were captured as direct BMP2-binding ligands. All six compounds promoted osteoblast differentiation and mineralization in MC3T3-E1 Subclone 14 cells, with IB displaying the strongest binding affinity and bioactivity. Mechanistically, IB failed to rescue the osteogenic suppression induced by the BMP type I receptor inhibitor LDN-193189, indicating its dependence on BMP signaling. In an LPS-induced inflammatory model, IB significantly reversed the downregulation of key proteins in the BMP2-Smad pathway (p-Smad1, Smad4, and Runx2) and osteogenic marker genes (*Osterix*, *COL1A1*, and *OCN*), demonstrating its capacity to restore osteogenic function under compromised conditions. Collectively, these findings establish that caffeoylquinic acid derivatives, particularly IB, function as BMP2-targeting osteogenic constituents of *P. forrestii* that activate BMP2-Smad signaling to promote osteoblast differentiation, thereby offering a pharmacological basis for the development of natural product-derived osteogenic agents.

## Simple Summary

Bone loss and inflammatory joint diseases are major health challenges, creating a growing need for effective treatments from natural sources. *Periploca forrestii*, a plant used in traditional Miao medicine, has long been valued for treating bone-related conditions, yet its active components remain unclear. In this study, we used a modern screening technique to “fish out” the specific compounds from this plant that bind to a key protein involved in bone formation. We successfully identified six natural compounds, among which isochlorogenic acid B showed the strongest activity. These compounds were tested in bone cell cultures and were found to promote osteoblast differentiation and mineralization. In particular, isochlorogenic acid B was able to restore bone-forming function even under conditions that mimic inflammation. Our findings reveal that isochlorogenic acid B and related compounds work by activating a specific cellular signaling pathway (BMP2-Smad) to promote bone formation. This research provides a scientific foundation for developing natural, plant-based agents that could support bone health and help counteract bone destruction in inflammatory diseases, offering a potential alternative to conventional therapies.

## 1. Introduction

The BMP2-Smad signaling pathway is a central driver of osteoblast differentiation and bone formation [1]. Binding of BMP2 to its receptors triggers Smad1/5/8 phosphorylation, association with Smad4, nuclear translocation, and transcriptional activation of osteogenic genes such as Runx2 and Osterix, promoting proliferation, differentiation, and mineralization [2,3,4]. Given its pivotal role in bone formation, the BMP2-Smad pathway represents an attractive target for discovering osteogenic agents capable of enhancing bone formation and restoring osteogenic function under pathological conditions [5,6].

Natural products derived from medicinal plants offer a valuable source of small-molecule BMP2 ligands with osteogenic potential. Surface plasmon resonance (SPR)-based target fishing provides a powerful label-free approach for directly capturing ligands that bind to a target protein from complex herbal extracts [7], facilitating the discovery of bioactive compounds that activate the BMP2-Smad pathway. *Periploca forrestii*, a traditional Miao medicinal plant used for bone-related conditions, has been reported to promote osteogenesis [8,9]. Our previous studies [10], integrating serum pharmacochemistry, network pharmacology, and transcriptomics, demonstrated that *P. forrestii* enhances osteoblast activity and promotes bone formation through activation of the BMP-Smad pathway. Molecular docking analyses further revealed strong binding affinities between the serum-available constituents of *P. forrestii* and the BMP2 target, suggesting that certain components, particularly caffeoylquinic acid derivatives, may exert osteogenic effects through direct interaction with BMP2. However, it remains unclear which specific serum-available monomers directly bind to BMP2 protein and whether these monomers promote osteoblast differentiation via activation of the BMP2-Smad pathway. Importantly, given that inflammation-induced suppression of osteoblast function is a key contributor to bone loss in conditions such as rheumatoid arthritis, identifying BMP2-targeting compounds with the ability to restore osteogenic function under inflammatory conditions holds significant therapeutic potential.

Based on these findings, we hypothesize that the osteogenic constituents of *P. forrestii* exert their effects through direct binding to BMP2 and subsequent activation of the downstream Smad signaling cascade, thereby promoting osteoblast differentiation and restoring osteogenic capacity under compromised conditions. To test this hypothesis, the present study employed SPR-based target fishing against BMP2 to screen for potential BMP2-binding ligands from *P. forrestii*. The identified compounds were further evaluated for their effects on BMP-Smad pathway activation, osteogenic gene expression, and osteoblastic function in MC3T3-E1 Subclone 14 cells, a well-established in vitro model for studying osteoblast differentiation. This study aims to identify the pharmacodynamic material basis and elucidate the molecular mechanism by which *P. forrestii* promotes osteogenesis through BMP2-dependent pathways, thereby providing a pharmacological foundation for the discovery of BMP2-targeting osteogenic agents from natural products.

## 2. Materials and Methods

### 2.1. SPR-Based Fishing of BMP2 Ligands from the Ethanolic Extract of Periploca forrestii

#### 2.1.1. Preparation of Test Sample

The preparation of the ethanol extract of *Periploca forrestii* was carried out based on the previously reported method [10]. A 10 mg aliquot of the ethanolic extract of *P. forrestii* was dissolved in 5% DMSO buffer and PBST, with vortexing and ultrasonication to ensure complete dissolution, yielding a 10 mg/mL test solution for subsequent use.

#### 2.1.2. Immobilization of BMP2 Protein

**Figure 1:**
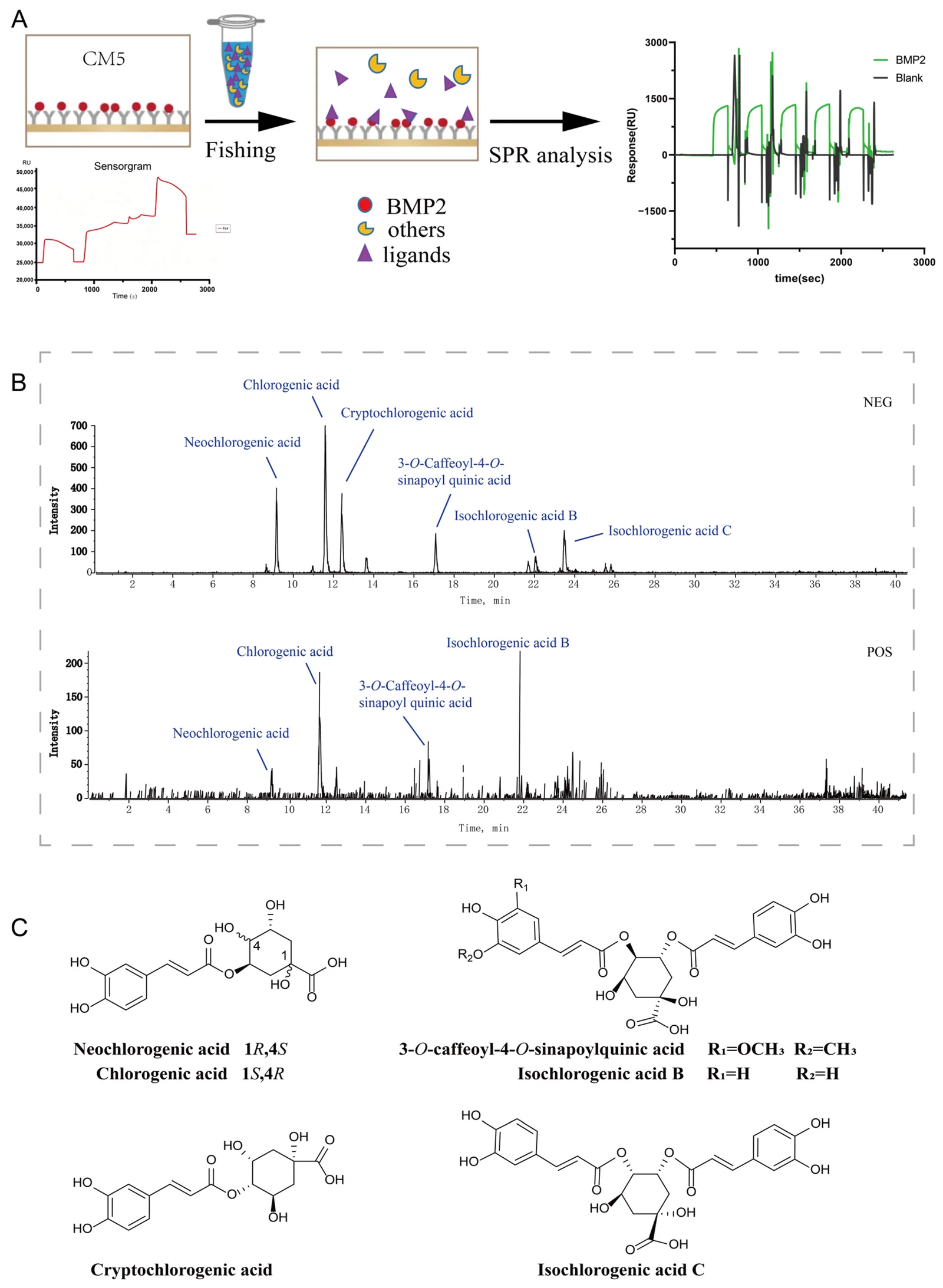
SPR-based target fishing against BMP2 from the ethanolic extract of *P. forrestii*. (**A**) Schematic diagram of the fishing procedure. (**B**) UPLC-Q-TOF-MS analysis of the BMP2 fishing eluate. (**C**) Chemical structures of the six identified BMP2-binding ligands.

The BMP2 protein was immobilized onto a CM5 sensor chip using the manual mode. The immobilization procedure comprised three consecutive steps: (i) chip activation, in which 100 µL of NHS and 100 µL of EDC were mixed and injected at a flow rate of 10 µL/min for 420 s; (ii) protein immobilization, performed at a flow rate of 10 µL/min with a protein concentration of 50 µg/mL for 600 s or longer, using Acetate 4.0 (BR100349, Cytiva, Uppsala, Sweden) for protein dilution; and (iii) chip blocking, achieved by injecting ethanolamine at 10 µL/min for 420 s using the Amine Coupling Kit (BR100050, Cytiva, Uppsala, Sweden). The immobilization level of BMP2 reached 8000 resonance units (RU) (Figure 1A).

#### 2.1.3. Fishing Procedure

The SPR fishing program was set to the “inject and recover” mode, with the following parameters: contact time 180 s; dissociation time 200 s; flow rate 30 µL/min; temperature 25 °C. The ethanolic extract of *P. forrestii* (10 mg/mL) was sequentially filtered through 0.5 µm and 0.22 µm membranes prior to loading onto the instrument. This concentration was selected based on preliminary SPR screening to ensure a sufficient signal-to-noise ratio while minimizing non-specific binding. Five injections were collected per cycle, and a total of 10 cycles were performed to accumulate adequate ligand quantities for subsequent UPLC-Q-TOF-MS identification. A blank control group was included, using a chip without immobilized protein for fishing.

#### 2.1.4. Structural Characterization of Fished Compounds

The fishing eluates from both the blank control and BMP2 groups were collected and analyzed by UPLC-Q-TOF-MS (Waters H-Class Ultra Performance Liquid Chromatography, Waters Technologies Corporation; AB Sciex Triple TOF® 4600 High-Resolution Mass Spectrometer, SCIEX Corporation) for mass spectrometric data acquisition [10]. Chemical components were identified by comparison with literature reports and the Standard Natural Products High-Resolution Mass Spectral Database (Shanghai Standard Technology Co., Ltd., Shanghai, China), and further confirmed by co-chromatography with reference standards from the same company. Combined with literature data and the identification results of the original extract, the compounds that bound to BMP2 protein in *P. forrestii* were conclusively determined. The proportion and gradient of the mobile phase are shown in Table S1. Mass spectrometry detection was conducted in ESI-negative/positive ion mode, with parameters provided in Table S2.

### 2.2. Molecular Docking

Molecular docking was conducted with AutoDockTools (v1.5.7). to investigate the interaction between neochlorogenic acid (NCA), chlorogenic acid (CA), cryptochlorogenic acid (CCA), isochlorogenic acid B (IB), and isochlorogenic acid C (IC). The BMP2 protein (PDBID: 4ui1) was obtained from the RCSB Protein Data Bank (RCSB PDB, https://www.rcsb.org). The three-dimensional structure of chemical compounds was generated using Chem3D 22.0 software. Protein-compound docking was carried out using the vinna program, hydrogen atoms were added to the protein structures, and all water molecules were eliminated using AutoDockTools (v1.5.7). PyMOL(TM) 3.1.3 software was then used to visualize the docking results. Chlorogenic acid, a well-recognized bioactive constituent reported to modulate the BMP-related osteogenic pathways in bone metabolism studies, was selected as a positive reference ligand for comparative docking analysis [11].

### 2.3. Cell Culture and Differentiation

Mouse calvarial preosteoblast subclone 14 cells (MC3T3-E1 Subclone 14, Servicebio, Wuhan, China, Cat. No. STCC20026P-1) were purchased from Wuhan Servicebio Technology Co., Ltd. The cells were resuspended by gentle pipetting in an appropriate volume of complete medium (α-MEM supplemented with 10% fetal bovine serum) and seeded into gas-permeable cell culture flasks. Cells were maintained at 37 °C in a humidified atmosphere containing 5% CO_2_. To induce osteogenic differentiation, the culture medium was replaced with osteogenic induction medium (Servicebio, Wuhan, China, G4119).

### 2.4. Cell Viability Assay

MC3T3-E1 Subclone 14 cells at the logarithmic growth phase were harvested by trypsinization and resuspended in complete medium. The cells were seeded into 96-well plates at a density of 5 × 10^3^ cells per well and incubated at 37 °C in a 5% CO_2_ atmosphere for 24 h. The culture medium was then discarded, and the cells were treated with fresh medium containing various concentrations (200, 100, 50, 25, 12.5, and 6.25 µM) of neochlorogenic acid (NCA), chlorogenic acid (CA), cryptochlorogenic acid (CCA), isochlorogenic acid B (IB), and isochlorogenic acid C (IC), respectively. After further incubation for 48 h, the medium was removed, and the cells were washed once with PBS. Subsequently, 100 µL of α-MEM medium containing 10% CCK-8 reagent was added to each well, and the plates were incubated for an additional 1–2 h. The absorbance of each well was measured at 450 nm using a microplate reader. Cell viability was calculated, and dose–response curves were generated accordingly.

$$Relative\;cell\;viability\;(\%)=\frac{OD_{experimental}-OD_{blank}}{OD_{control}-OD_{blank}}\times 100\%$$

The control group (usually untreated cells) was set as 100%, and all data from experimental groups were normalized to this baseline.

### 2.5. ALP Staining and Quantitative Analysis

MC3T3-E1 Subclone 14 cells were seeded into 24-well plates at a density of 2.5–3 × 10^4^ cells per well and cultured for 24 h to reach 70–80% confluence. The culture medium was then replaced with 500 µL of osteogenic induction medium per well in the CON group, while cells in the experimental groups received 500 µL of osteogenic induction medium containing the appropriate concentrations of the test compounds. After 48 h of incubation, the medium was refreshed with blank osteogenic induction medium every 2–3 days. On day 7, the culture medium was aspirated, and the cells were washed three times with PBS (3–5 min each wash), followed by fixation with 40 g/L neutral formaldehyde for 20 min. After three additional washes with PBS (10 min each), the BCIP/NBT working solution (Beyotime, Shanghai, China, C3206) was prepared according to the manufacturer’s instructions. An aliquot of 250 µL of the working solution was added to each well, and the plates were incubated in the dark until purple granules were visible to the naked eye. The cells were then rinsed 2–3 times with deionized water and air-dried. Images were captured under an inverted microscope. For quantitative analysis, the culture medium in parallel wells was discarded, and the cells were lysed with 100 µL of cell lysis buffer per well. The ALP activity was determined according to the manufacturer’s protocol of the alkaline phosphatase assay kit (Beyotime, Shanghai, China, P0321S), and the absorbance of each well was measured at 405 nm using a microplate reader. The ALP content was subsequently calculated. All experiments were performed in triplicate.

### 2.6. Alizarin Red S (ARS) Staining and Quantitative Analysis

Cell seeding, culture, and drug treatment were performed as described in Section 2.5. On day 21, the culture medium was aspirated, and the cells were gently washed three times with PBS (3–5 min each wash), followed by fixation with 40 g/L neutral formaldehyde for 20 min. After three gentle washes with PBS (10 min each), 250 µL of Alizarin Red S staining solution (OriCell, Shanghai, China, ALIR-10001) was added to each well and incubated for 5–10 min. The cells were then gently rinsed 2–3 times with deionized water and air-dried. Images were captured under an inverted microscope. Following drying of the plates, 350 µL of 10% cetylpyridinium chloride solution was added to each well to solubilize the mineralized nodules. The absorbance of each well was measured at 450 nm using a microplate reader, and the relative calcium nodule content was calculated. All experiments were performed in triplicate.

### 2.7. Cell Scratch (Wound Healing) Assay

Guide lines were marked on the underside of the plates using a marker pen to assist scratch alignment. MC3T3-E1 Subclone 14 cells were seeded into 6-well plates at a density of 1.5–2 × 10^5^ cells per well and cultured for 24 h until reaching 80–90% confluence. A straight scratch was then created through the cell monolayer using a 200 µL pipette tip perpendicular to the central axis of the well. Detached cells were removed by washing 2–3 times with PBS. After aspiration of PBS, an appropriate volume of serum-free basal medium containing the test compounds was added to each well. Images of the scratch gap at the same position were captured under an inverted microscope immediately (0 h) and at 12 h, 24 h, and 48 h post-scratching. The scratch areas were measured using ImageJ 1.54g software, and cell migration rates were calculated. All experiments were performed in triplicate. The cell migration rate was calculated using the following formula (N = 12, 24, and 48 h):

$$Cell\;migration\;rate\;(\%)=\frac{Scratch\;area\;at\;0\;h-Scratch\;area\;at\;N\;h}{Scratch\;area\;at\;0\;h}\times 100$$

### 2.8. Real-Time Quantitative PCR (RT-qPCR)

Cells were seeded, treated, and cultured as described in Section 2.5. After 7 days of culture, the old medium was aspirated, and the cells were rinsed 2–3 times with PBS. Subsequently, 350 µL of lysis buffer was added to each well, and total RNA (*n* = 3 wells per group) was extracted strictly following the manufacturer’s protocol of the RNA extraction kit (SparkJade, Jinan, China, lot: WVJWL), with concentration and purity assessed via agarose gel electrophoresis and spectrophotometry (Implen N60-Touch, Munich, Germany). Reverse transcription was performed using a SparkJade kit, Jinan, China (lot: WFVSX) to generate cDNA. RT-qPCR amplification comprised: initial denaturation at 95 °C for 10 min; 45 cycles of denaturation (95 °C, 15 s) and combined annealing/extension (60 °C, 60 s). Gene expression levels were quantified using the 2^−ΔΔCt^ method. The target genes (*Bmp-2*, *Smad1*, *Smad4*, *Smad5*, *Smad8*, *Runx2*, *COL1A*, *OCN* and *Osterix*) were normalized to *GAPDH*. Primer sequences are provided in Table S3.

### 2.9. Western Blotting (WB)

Cells were seeded, treated, and cultured as described in Section 2.5. After 7 days of culture, the osteogenic induction medium was aspirated and discarded, and the cells were washed with PBS. Subsequently, 100 µL of lysis buffer was added to each well to extract total cellular protein. Protein concentration was determined using a BCA protein assay kit (Beyotime, Shanghai, China), with an approximate loading amount of 30 µg per sample. Western blot analysis (*n* = 3 wells per group) was performed using the following primary antibodies: Smad1 (1:3000, 66559-1-Ig, Proteintech, Wuhan, China), p-Smad1 (1:1000, HA722566, HUAbio, Hangzhou, China), Smad4 (1:1000, PB0446, BOSTER, Wuhan, China), Runx2 (1:1000, No.F2885, Selleck, Shanghai, China) and anti-β-actin (1:10,000, ET1702-67, HUAbio, Hangzhou, China). A HRP-conjugated goat antirabbit IgG secondary antibody (1:10,000, YP848537-H, UpingBio, Hangzhou, China) was used for detection.

### 2.10. Statistical Analysis

Image J 1.54g software was used to analyze the expression of Smad1, p-Smad1, Smad4 and Runx2 in the MC3T3-E1 Subclone 14 cells. Data normality was assessed using the Shapiro–Wilk test, and homogeneity of variance was evaluated with Levene’s test. For normally distributed data with equal variance, one-way ANOVA followed by Tukey’s post hoc test was applied. Non-parametric Kruskal–Wallis test with Dunn’s correction was used when assumptions were not met. Statistical analysis and graphing were performed using SPSS 26.0 and GraphPad Prism 9.5. Each experiment was repeated at least three times. *p* < 0.05 was considered statistically significant.

## 3. Results

### 3.1. Identification of BMP2 Ligands from Periploca forrestii

**Figure 2:**
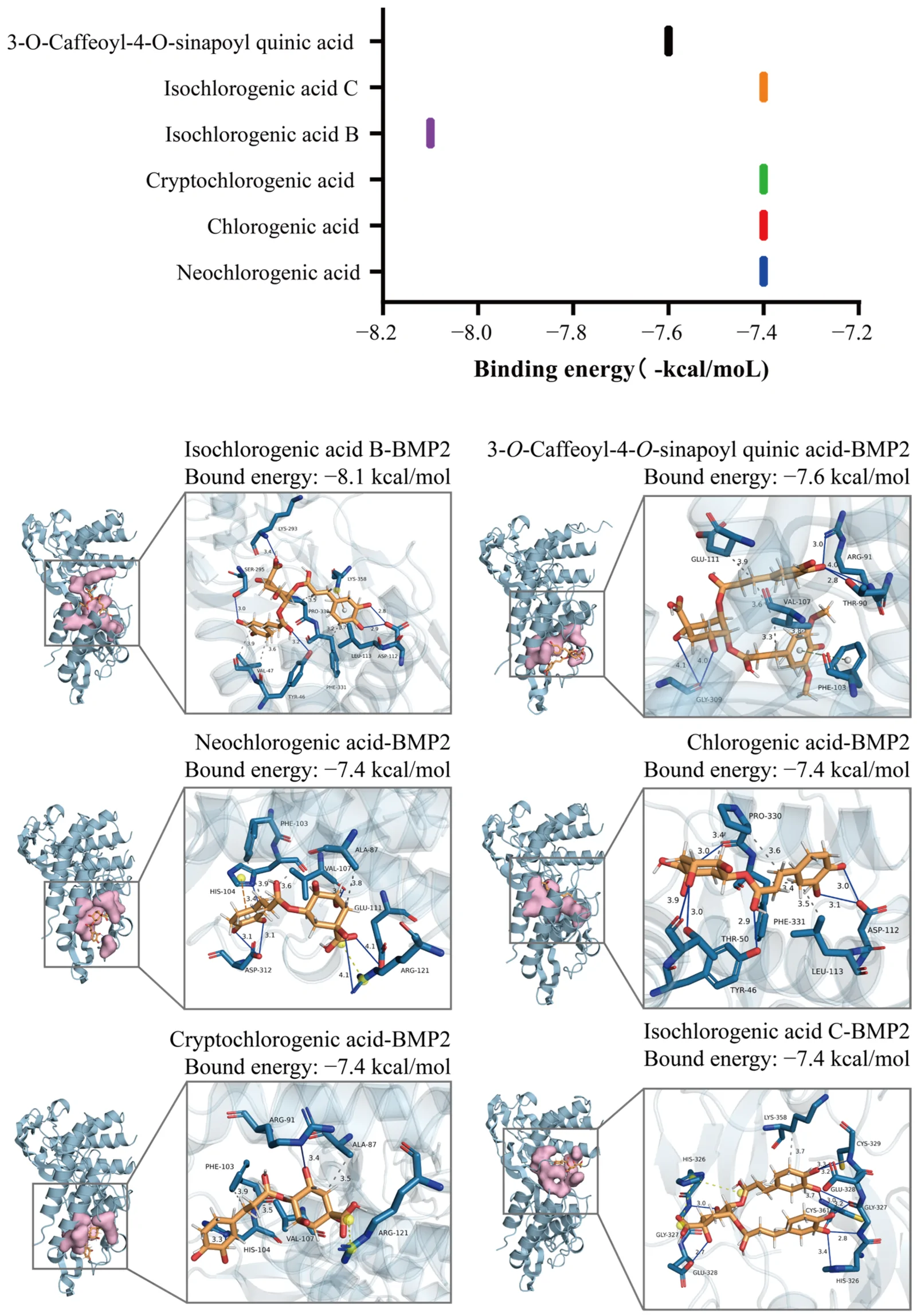
Molecular docking of BMP2 with potential active compounds.

SPR sensorgram analysis (Figure 1A) revealed a distinct specific binding signal in the BMP2 experimental group, as indicated by the corresponding green curve. This signal demonstrated specific interactions between the BMP2 protein immobilized on the chip surface and substances present in the reaction system, providing clear and direct visual evidence of the binding event. To definitively identify the ligand components that specifically bound to BMP2 protein, the binding components in the fishing eluates were systematically analyzed in conjunction with the previously established compositional profiling of the crude *P. forrestii* extract and the identification of serum-available components. Ultimately, six binding ligand components were identified from the fishing eluate samples (Figure 1B,C, Table S4), namely neochlorogenic acid (NCA), chlorogenic acid (CA), cryptochlorogenic acid (CCA), 3-*O*-caffeoyl-4-*O*-sinapoylquinic acid, isochlorogenic acid B (IB), and isochlorogenic acid C (IC) (Figures S1–S6) [12,13]. As previously shown by our research group through peak area ranking of the chemical constituents from the ethanolic extract of *P. forrestii* and the serum-available component profile, these compounds ranked among the top 10 in content, representing the major constituents of the extract and suggesting their potential as osteoprotective active constituents. Molecular docking results (Figure 2) demonstrated that all six ligands obtained by SPR-based fishing exhibited strong binding affinities to BMP2, with IB showing the most favorable binding energy (−8.1 kcal/moL).

### 3.2. Safety Evaluation and Osteogenic Activity of Potential Active Constituents in MC3T3-E1 Subclone 14 Cells

**Figure 3:**
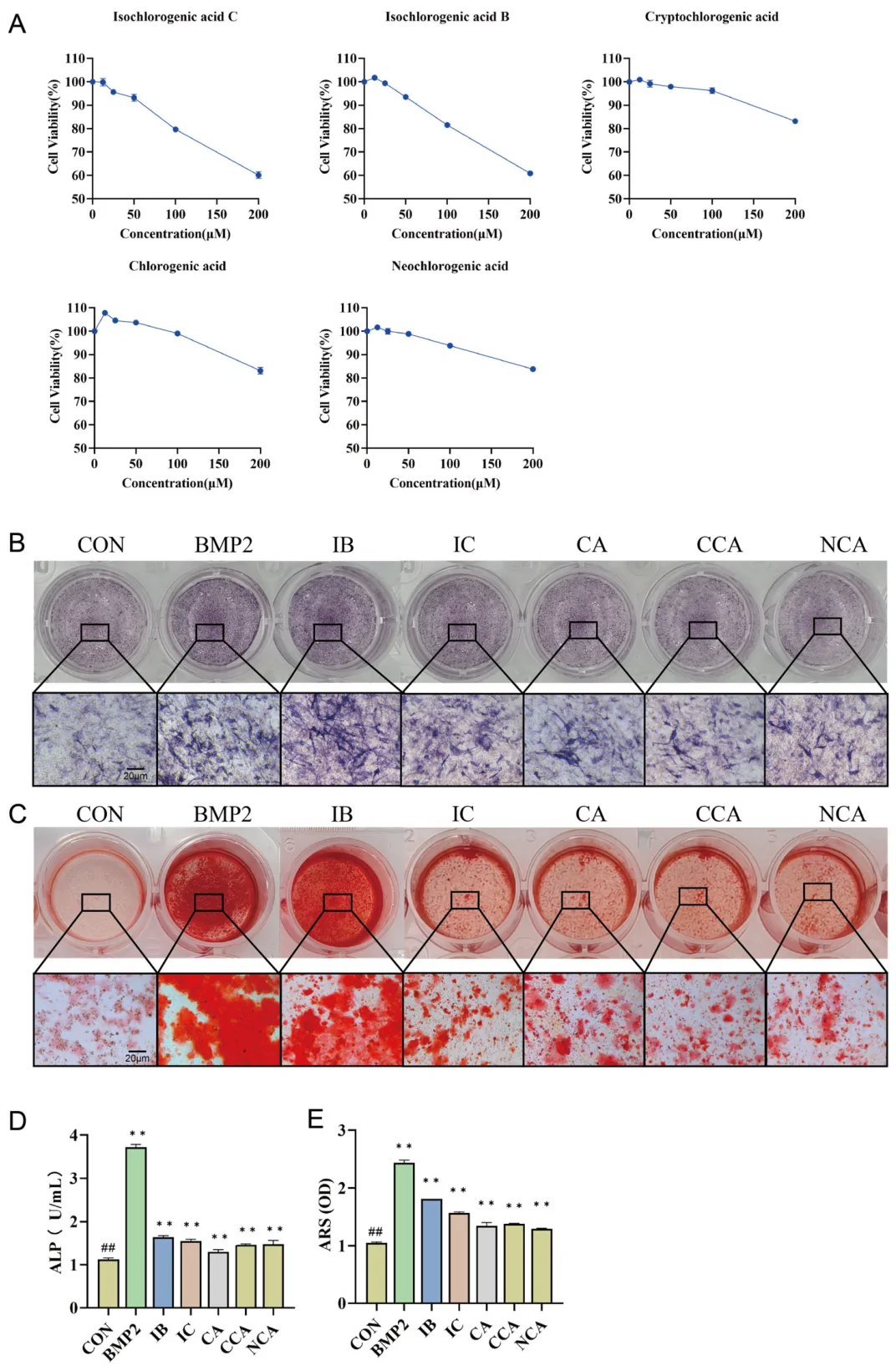
Effects of BMP2-binding ligands on MC3T3-E1 Subclone 14 cells. (**A**) Cell viability assessment after 48 h treatment with various concentrations of the potential active compounds. (**B**) ALP staining of potential active compounds (25 µM) on day 7. (**C**) ARS staining of potential active compounds (25 µM) on day 21. (**D**) Quantitative analysis of ALP activity. (**E**) Quantitative analysis of mineralized nodule formation. vs. CON, ** *p* < 0.01; CON vs. BMP2, ^##^ *p* < 0.01. Data are presented as mean ± SD (*n* = 3 independent experiments).

To determine a safe dose range for subsequent pharmacodynamic studies, the effects of the five test compounds (CA, CCA, NCA, IB, and IC) on cell viability were assessed. MC3T3-E1 Subclone 14 cells were treated with each compound at concentrations of 200, 100, 50, 25, and 12.5 µM for 48 h. Relative cell viability remained above 95% at concentrations up to 50 µM for all tested compounds, indicating no significant cytotoxicity. Notably, at 12.5 µM, the relative cell viability significantly increased to approximately 120–130% of the control, suggesting a potential pro-proliferative effect (Figure 3A). In accordance with the recommendation that compound concentrations in preclinical drug development should preferably remain within 50 µM, all subsequent pharmacodynamic experiments were conducted with doses not exceeding this threshold.

On this basis, the osteogenic activity of the five compounds was evaluated at a concentration of 25 µM, with recombinant BMP2 protein (50 ng/mL) serving as a positive control [14]. ALP and ARS staining revealed that, compared with the CON group, both recombinant BMP2 protein and each of the five compounds significantly promoted osteoblast differentiation and mineralization (Figure 3B–E). Among all tested compounds, IB exhibited the strongest effect, which was consistent with the molecular docking results. Therefore, IB was selected as the representative osteogenic active constituent from *P. forrestii* for subsequent mechanistic investigations. Based on the dose–response and safety evaluation, concentrations of 12.5, 25, and 50 µM were selected for IB in all subsequent experiments, representing low, medium, and high doses, respectively.

### 3.3. Effect of Isochlorogenic Acid B on Scratch Wound Healing in MC3T3-E1 Subclone 14 Cells

**Figure 4:**
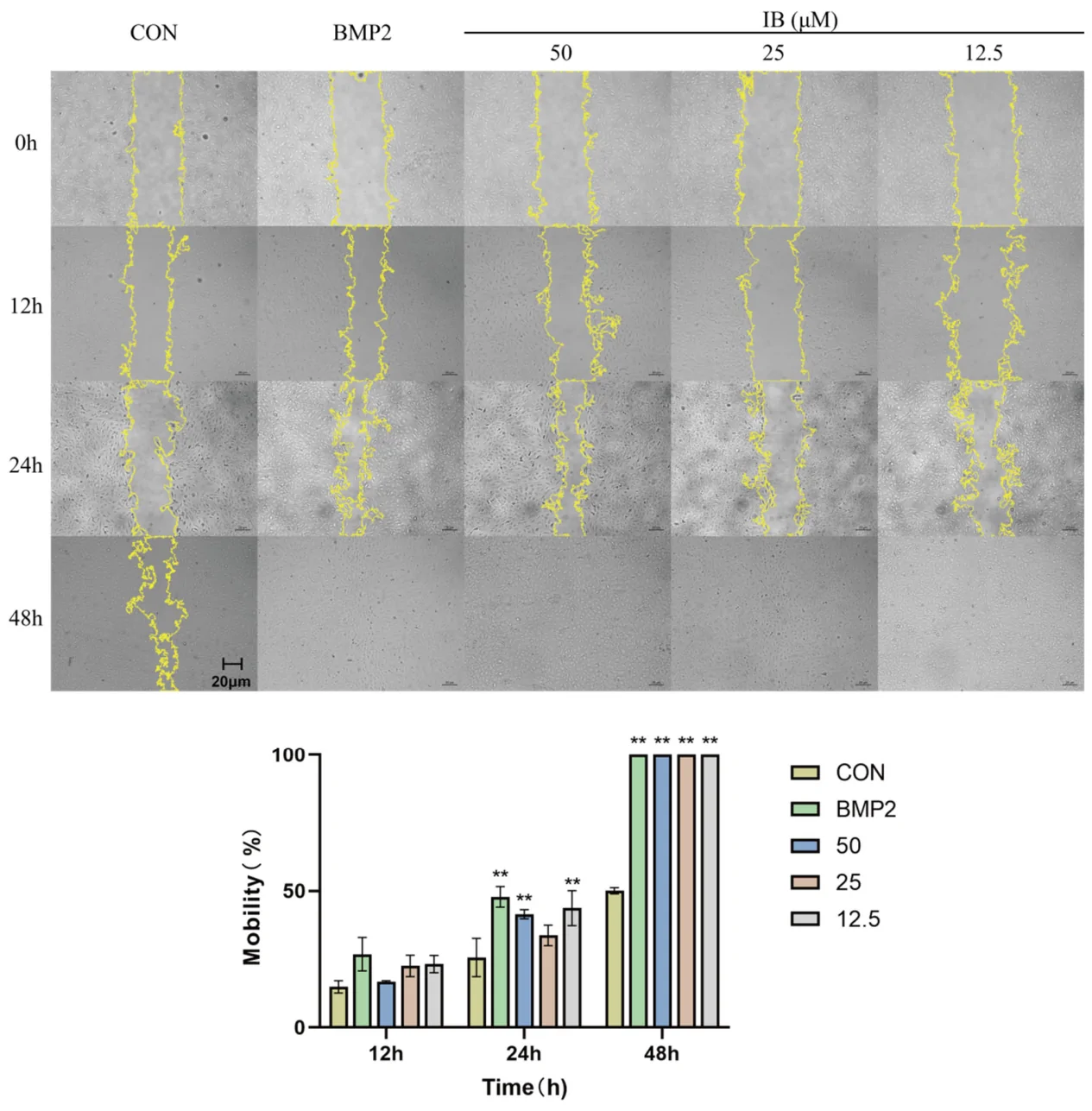
Effect of IB on wound healing in MC3T3-E1 Subclone 14 cells. Representative images and quantitative analysis of cell migration rates at 0, 12, 24, and 48 h. Yellow lines indicate the wound edges. vs. CON, ** *p* < 0.01. Data are presented as mean ± SD (*n* = 3 independent experiments).

The migratory capacity of MC3T3-E1 Subclone 14 cells was evaluated using a scratch wound healing assay. Photographs of the scratch gaps were captured at 0, 12, 24, and 48 h post-scratching. The scratch areas were measured using ImageJ software, and cell migration rates were calculated accordingly. The results demonstrated that, at 24 h post-scratching, the migration rate of cells in the treatment groups showed a statistically significant difference compared with the CON group. By 48 h, complete wound closure was observed in both the recombinant BMP2 protein group and all IB concentration groups (Figure 4).

### 3.4. Osteogenic Effects of IB on MC3T3-E1 Subclone 14 Cells

**Figure 5:**
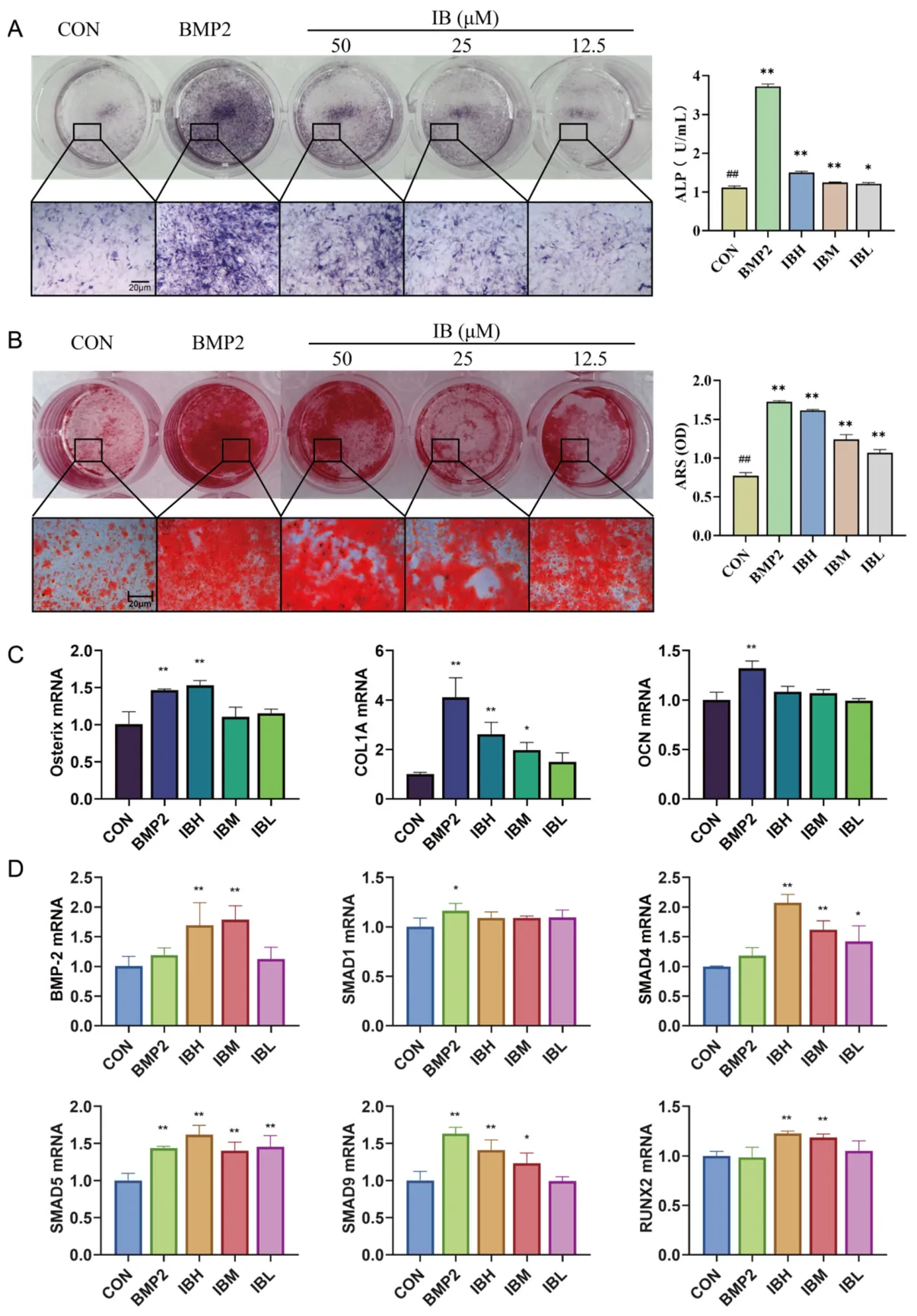
IB promotes osteogenesis and activates BMP2-Smad signaling in MC3T3-E1 Subclone 14 cells. (**A**) ALP staining and quantification. (**B**) ARS staining and quantification. (**C**) mRNA levels of osteogenic markers (*Osterix*, *COL1A1*, and *OCN*). (**D**) mRNA levels of BMP2-Smad pathway genes (*Bmp-2*, *Smad1*, *Smad4*, *Smad5*, *Smad8*, and *Runx2*). vs. CON, ** *p* < 0.01, * *p* < 0.05; CON vs. BMP2, ^##^ *p* < 0.01. Data are presented as mean ± SD (*n* = 3 independent experiments).

To evaluate the osteogenic effects of IB, cells were treated with IB at high, medium, and low doses (IBH, IBM, and IBL at 50, 25, and 12.5 µM, respectively), with recombinant BMP2 protein (50 ng/mL) serving as a positive control. On day 7, ALP staining was performed to assess the effect of IB on osteoblast differentiation, and on day 21, ARS staining was conducted to evaluate its effect on osteoblast mineralization. The results (Figure 5A,B) demonstrated that both recombinant BMP2 protein and IB significantly promoted osteoblast differentiation and mineralization. Furthermore, RT-qPCR analysis revealed that, compared with the CON group, IB upregulated the mRNA expression of the osteogenic marker genes *Osterix*, *COL1A1*, and *OCN*, indicating that IB exhibited considerable osteogenic potential (Figure 5C). To determine whether IB exerts its osteogenic effects through BMP signaling, the expression of key proteins and genes in the BMP2-Smad signaling pathway was assessed by RT-qPCR. RT-qPCR results (Figure 5D) demonstrated that IB intervention upregulated the mRNA expression of *Bmp-2*, *Smad1*, *Smad4*, *Smad5*, *Smad8*, and *Runx2* compared with the CON group.

### 3.5. Osteogenic Effects of Isochlorogenic Acid B Depend on the BMP2-Smad Signaling Pathway

LDN-193189, a potent and selective inhibitor of BMP type I receptors, is widely used in studies of BMP signaling. To determine its safe concentration range, MC3T3-E1 Subclone 14 cells were treated with LDN-193189 at concentrations of 0.3125, 0.625, 1.25, 2.5, 5, and 10 µM for 48 h, and cell viability was assessed by the CCK-8 assay (Figure S7). Cell viability remained above 80% at concentrations up to 1.25 µM. Based on these toxicity results and in accordance with reference [14], a concentration of 1 µM was selected for subsequent mechanistic validation.

Cells were seeded in 24-well plates and divided into the following experimental groups: blank control (CON); positive control (BMP2, rhBMP2 protein 50 ng/mL); inhibitor group (LDN, LDN-193189 1 µM); rhBMP2 protein following inhibitor intervention (LDB, 50 ng/mL); and IB high-, medium-, and low-dose groups following inhibitor intervention (LDH, LDM, and LDL at 50, 25, and 12.5 µM, respectively). For inhibitor experiments, cells were co-treated with LDN-193189 and IB at the indicated concentrations for 48 h at the onset of osteogenic induction. On day 7, ALP staining and RT-qPCR analysis of osteogenic marker genes (*Osterix*, *COL1A1*, and *OCN*) were performed, while ARS staining was conducted on day 21.

**Figure 6:**
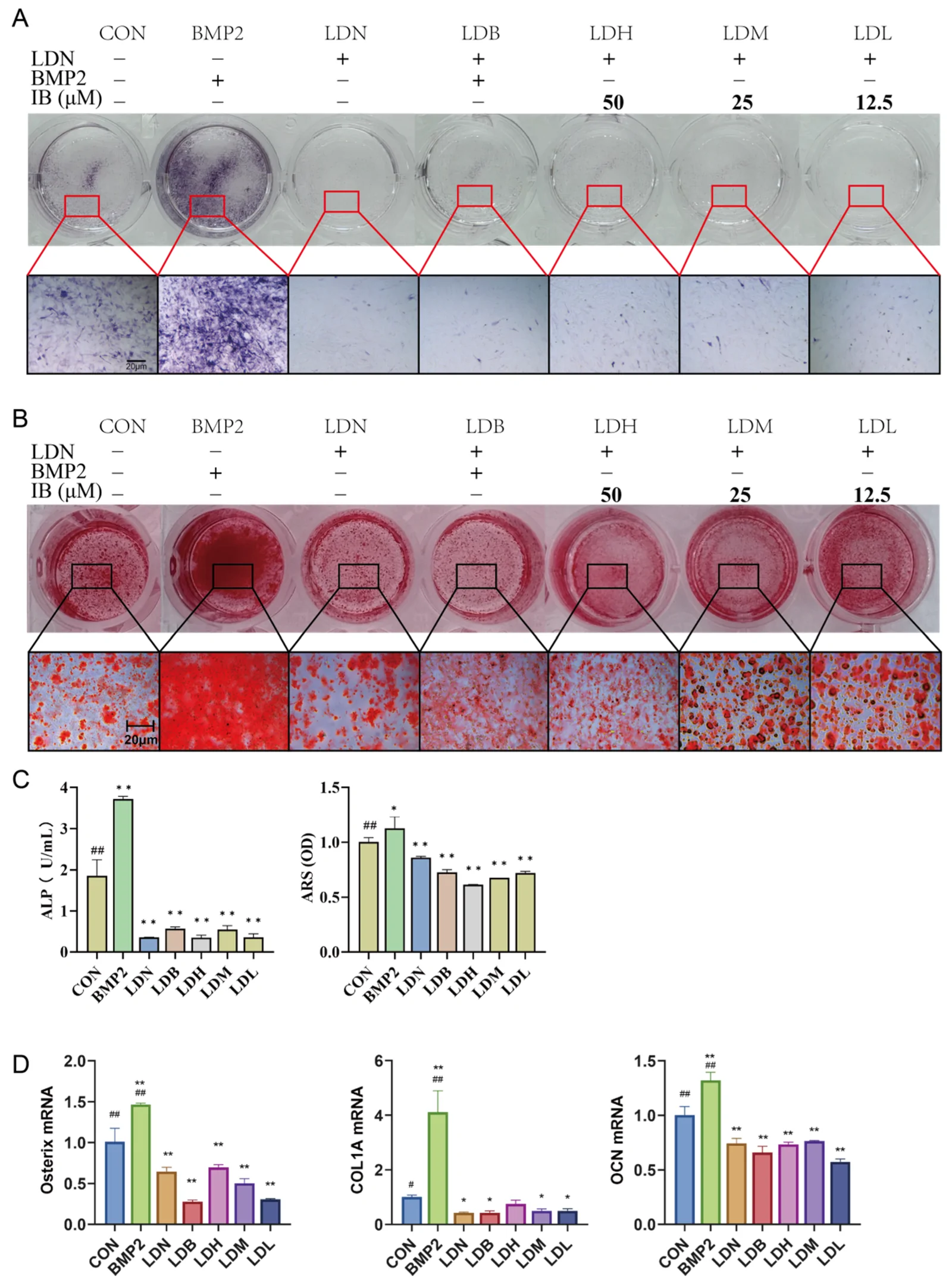
Effects of isochlorogenic acid B (IB) on LDN-193189-treated MC3T3-E1 Subclone 14 cells. (**A**) ALP staining on day 7. (**B**) ARS staining on day 21. (**C**) Quantitative analysis of ALP activity and mineralized nodule formation. (**D**) mRNA expression of osteogenic marker genes *Osterix*, *COL1A1*, and *OCN* determined by RT-qPCR. vs. CON, ** *p* < 0.01, * *p* < 0.05; vs. LDN, ^#^ *p* < 0.05, ^##^ *p* < 0.01. Data are presented as mean ± SD (*n* = 3 independent experiments).

As shown in Figure 6A–C, the recombinant BMP2 protein positive control significantly promoted osteoblast differentiation and mineralization compared with the CON group, whereas LDN-193189 effectively suppressed these processes. Notably, neither recombinant BMP2 protein nor IB at any concentration rescued the suppression induced by LDN-193189, preliminarily suggesting that IB promotes osteoblast differentiation in a BMP signaling-dependent manner. RT-qPCR results (Figure 6D) further demonstrated that IB upregulated the mRNA expression of *Osterix*, *COL1A1*, and *OCN*, while LDN-193189 downregulated these genes. IB treatment failed to reverse the LDN-193189-induced downregulation. To further determine whether IB exerts its osteogenic effects through the BMP signaling pathway, the expression of key genes and proteins in the BMP2-Smad cascade was examined.

**Figure 7:**
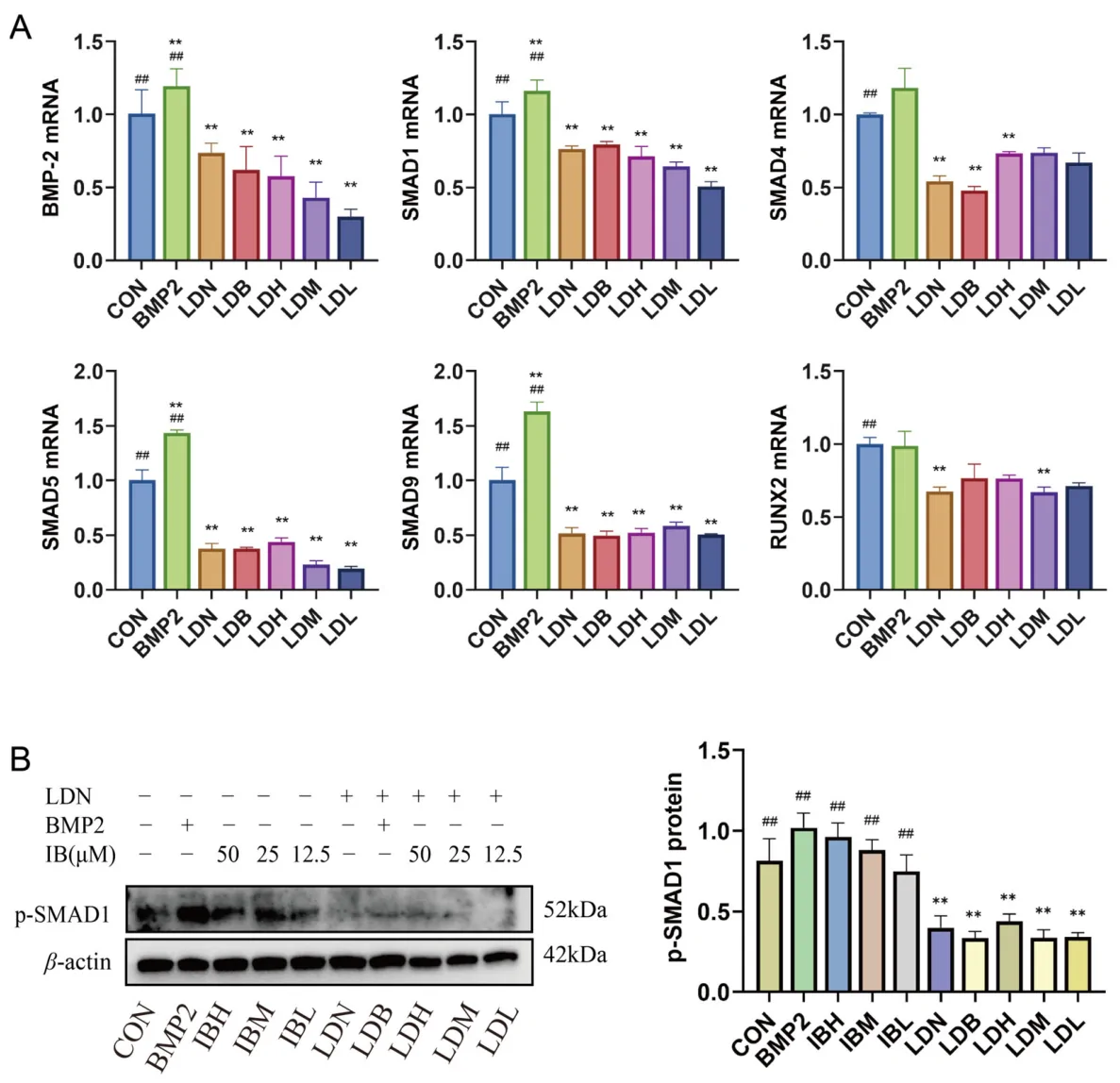
IB does not reverse LDN-193189-induced downregulation of BMP2-Smad pathway genes and p-Smad1. (**A**) mRNA levels of *Bmp-2*, *Smad1*, *Smad4*, *Smad5*, *Smad8*, and *Runx2*. (**B**) p-Smad1 protein levels. vs. CON, ** *p* < 0.01 vs. LDN, ^##^ *p* < 0.01. Data are presented as mean ± SD (*n* = 3 independent experiments).

RT-qPCR results (Figure 7A) showed that IB upregulated the mRNA expression of *Bmp-2*, *Smad1*, *Smad4*, *Smad5*, *Smad8*, and *Runx2*, whereas LDN-193189 significantly downregulated these genes, and IB failed to rescue the inhibitor-induced suppression.

Western blot analysis (Figure 7B and Figure S8) revealed that both recombinant BMP2 protein and IB upregulated the phosphorylation level of Smad1 (p-Smad1), a downstream effector of BMP2, while LDN-193189 decreased p-Smad1 expression. IB treatment did not significantly reverse the LDN-193189-induced downregulation of p-Smad1. Collectively, these findings suggest that the osteogenic effects of IB are dependent on the BMP2-Smad signaling pathway.

### 3.6. Osteogenic Effects of IB on LPS-Induced MC3T3-E1 Subclone 14 Cells

To evaluate whether IB exerts osteogenic effects under inflammatory conditions, the following experimental groups were established: control group (CON); inflammation model group (LPS, 0.5 µg/mL); rhBMP2 protein group following LPS intervention (LPB, 50 ng/mL); methotrexate group following LPS intervention (LPMT, 10 µM); and IB high-, medium-, and low-dose groups following LPS intervention (LPH, LPM, and LPL at 50, 25, and 12.5 µM, respectively). On day 7, ALP staining and RT-qPCR analysis of osteogenic marker genes (*Osterix*, *COL1A1*, and *OCN*) were performed, while ARS staining was conducted on day 21.

**Figure 8:**
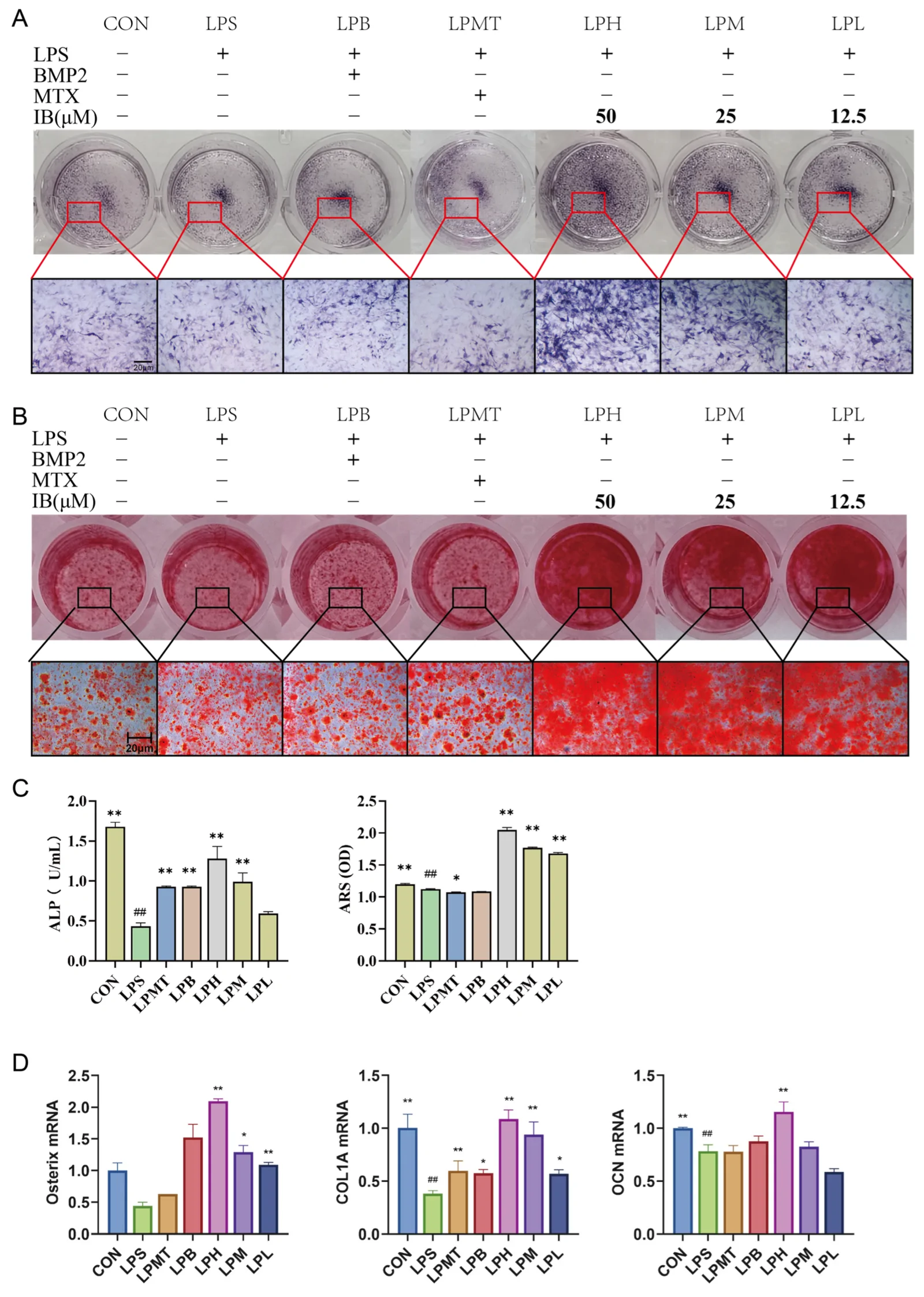
Effects of IB on LPS-treated MC3T3-E1 Subclone 14 cells. (**A**) ALP staining on day 7. (**B**) ARS staining on day 21. (**C**) Quantitative analysis of ALP activity and mineralized nodule formation. (**D**) mRNA expression of osteogenic marker genes *Osterix*, *COL1A1*, and *OCN* determined by RT-qPCR. vs. LPS, ** *p* < 0.01, * *p* < 0.05; LPS vs. CON, ^##^ *p* < 0.01. Data are presented as mean ± SD (*n* = 3 independent experiments).

As shown in Figure 8A–C, LPS alone did not markedly suppress osteoblast differentiation compared with the CON group. However, treatment with recombinant BMP2 protein following LPS stimulation failed to significantly promote osteoblast differentiation, preliminarily suggesting that LPS may inhibit osteogenic differentiation. In contrast, IB at all tested concentrations significantly promoted osteoblast differentiation and mineralization compared with the LPS group.

Further examination of osteogenic marker gene expression in the inflammatory microenvironment (Figure 8D) revealed that LPS downregulated the mRNA expression of *Osterix*, *COL1A1*, and *OCN* relative to the CON group, whereas IB treatment effectively restored the expression of these downregulated genes.

**Figure 9:**
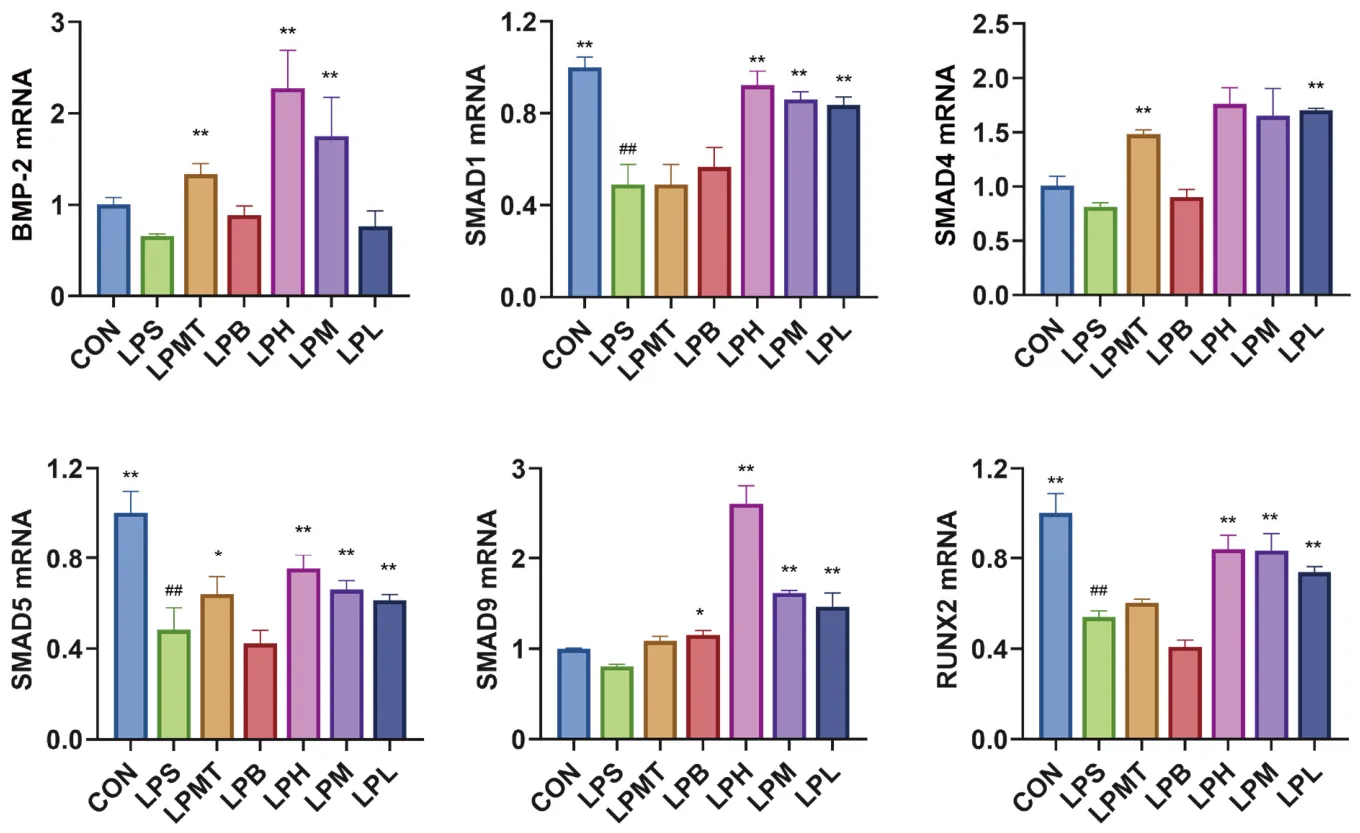
IB reverses LPS-induced suppression of BMP2-Smad signaling at the mRNA levels of *Bmp-2*, *Smad1*, *Smad4*, *Smad5*, *Smad8*, and *Runx2*. vs. LPS, ** *p* < 0.01, * *p* < 0.05; LPS vs. CON, ^##^ *p* < 0.01. Data are presented as mean ± SD (*n* = 3 independent experiments).

To determine whether IB exerts its osteogenic effects through BMP signaling even under inflammatory conditions, the expression of key proteins and genes in the BMP2-Smad signaling pathway was assessed by RT-qPCR and Western blotting. RT-qPCR results (Figure 9) demonstrated that LPS intervention downregulated the mRNA expression of *Bmp-2*, *Smad1*, *Smad4*, *Smad5*, *Smad8*, and *Runx2* compared with the CON group, while IB treatment significantly reversed these LPS-induced downregulations.

**Figure 10:**
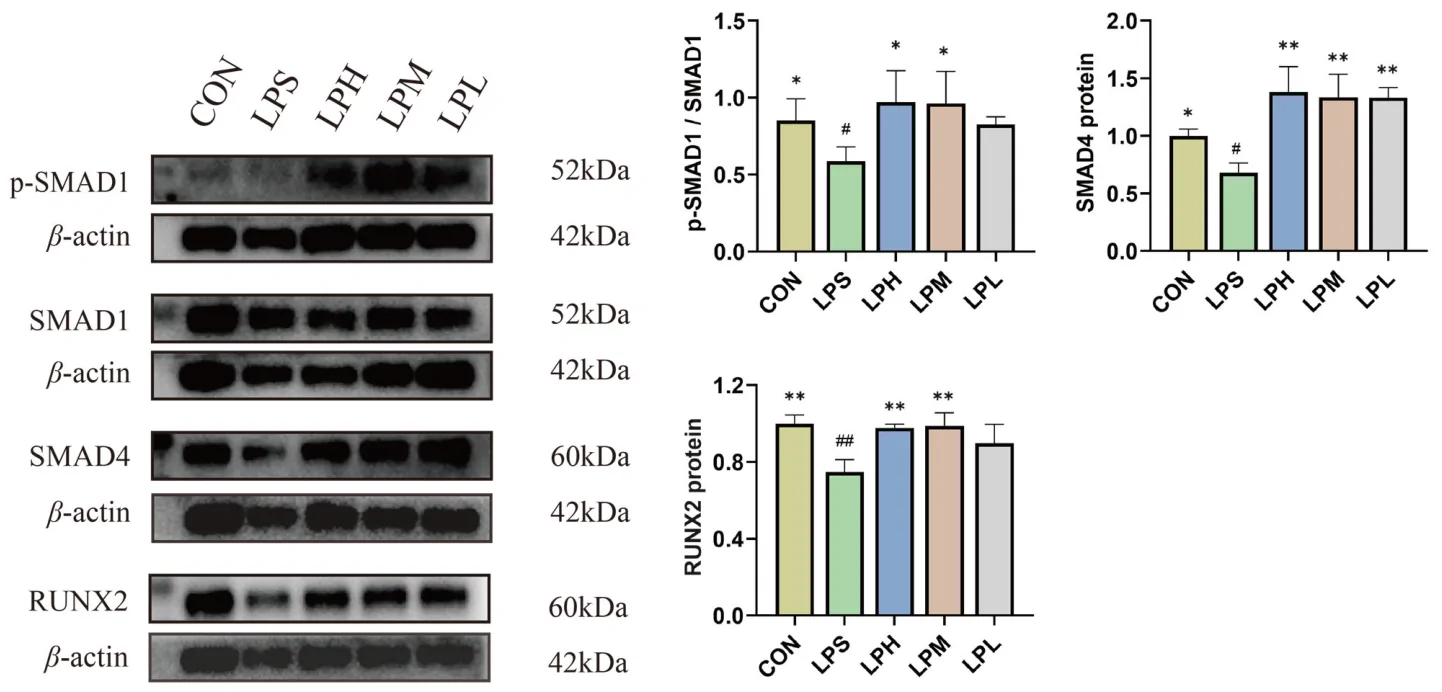
IB reverses LPS-induced suppression of BMP2-Smad signaling at the protein levels of Smad1, p-Smad1, Smad4, and Runx2. vs. LPS, ** *p* < 0.01, * *p* < 0.05; LPS vs. CON, ^##^ *p* < 0.01, ^#^ *p* < 0.05. Data are presented as mean ± SD (*n* = 3 independent experiments).

Western blot analysis (Figure 10 and Figures S9–S12) further demonstrated that, compared with the control group, LPS decreased the phosphorylation level of Smad1 (p-Smad1), a downstream effector of BMP2, while IB treatment upregulated p-Smad1 expression. Additionally, the levels of Smad4, which binds to phosphorylated Smad1, and the downstream transcription factor Runx2 were examined. The results showed that IB treatment restored the levels of these proteins, indicating that the LPS-induced suppression of the BMP2-Smad signaling pathway was reversed.

In the present experimental setting, LPS alone (0.5 µg/mL) did not significantly suppress ALP activity or mineralization compared with the CON group at the tested time points (Figure 8C). This is likely due to the relatively mild inflammatory challenge or the limited exposure duration, which may be insufficient to produce a robust inhibitory phenotype at the differentiation level. Nevertheless, LPS treatment did downregulate BMP2-Smad pathway genes and proteins (Figure 9 and Figure 10), indicating that the inflammatory insult was biologically active at the molecular level.

## 4. Discussion

In this study, surface plasmon resonance (SPR)-based target fishing was employed to screen for BMP2-binding constituents from the serum-available components of the Miao medicine *P. forrestii*. Six caffeoylquinic acid derivatives were identified as potential osteoprotective compounds, namely neochlorogenic acid (NCA), chlorogenic acid (CA), cryptochlorogenic acid (CCA), 3-*O*-caffeoyl-4-*O*-sinapoylquinic acid, isochlorogenic acid B (IB), and isochlorogenic acid C (IC) (Figure 1). These compounds were previously shown to rank among the top 10 constituents of *P. forrestii* in terms of peak area, indicating that they represent the major components of the extract [10]. This finding suggests that the pharmacological effects of *P. forrestii* are mediated by its principal constituents acting directly on key targets, a feature consistent with the characteristic “multi-component, multi-target” mode of action of traditional Chinese medicines, while also highlighting a “major component-core target” direct interaction paradigm.

Inflammatory conditions are characterized by elevated levels of pro-inflammatory cytokines such as TNF-α, IL-1β, and IL-17, which have been shown to directly suppress osteoblast differentiation. Additionally, neutrophil extracellular traps (NETs) have been reported to impede osteogenesis by activating the NF-κB pathway and downregulating BMP2 expression [15]. These observations indicate that inflammation-induced impairment of osteoblast function represents a common pathological mechanism across various bone loss conditions. Given that the BMP-Smad pathway is essential for maintaining bone mass and structural stability [16,17], identifying osteogenic agents capable of restoring BMP2-Smad signaling under inflammatory conditions is of considerable therapeutic relevance. This is particularly pertinent in diseases such as rheumatoid arthritis, where inflammation-driven bone destruction is a hallmark feature. In MC3T3-E1 Subclone 14 cells, all six identified BMP2 ligands exhibited osteogenic activity to varying degrees. Both ALP and ARS staining demonstrated that these compounds promoted osteoblast differentiation and subsequent mineralization. Among them, IB showed the strongest binding affinity to BMP2, as consistently revealed by molecular docking and SPR analyses. Further mechanistic investigation revealed that IB failed to reverse the impairment of osteogenic capacity under BMP signaling inhibition, suggesting that its pro-osteogenic effect is dependent on the BMP signaling pathway. In the inflammatory condition induced by LPS, stimulation of MC3T3-E1 Subclone 14 cells significantly downregulated the expression of key proteins in the BMP2-Smad signaling pathway, including p-Smad1, Smad4, and Runx2, whereas IB treatment effectively restored their expression levels (Figure 8, Figure 9 and Figure 10). The relatively mild suppression of ALP activity by LPS alone in our model may reflect the moderate concentration and duration of LPS exposure chosen to avoid excessive cytotoxicity. Despite this, the observed downregulation of BMP2-Smad signaling proteins and osteogenic genes confirmed that the inflammatory condition was functionally relevant. Importantly, IB treatment reversed these molecular changes, supporting its osteoprotective role under inflammatory stress.

The present findings connect the osteogenic effects of caffeoylquinic acid derivatives with previously reported bone-protective activities of chlorogenic acid and its analogues. Previous investigations have demonstrated that chlorogenic acid promotes osteogenic differentiation through multiple signaling pathways. Specifically, chlorogenic acid has been shown to facilitate osteogenic differentiation via the BMP signaling pathway, thereby preventing bone loss in ovariectomized mice [18,19,20]. In parallel, chlorogenic acid promotes osteogenic differentiation of human dental pulp stem cells through activation of the Wnt signaling pathway [21]. Beyond its differentiation-promoting effects, chlorogenic acid also exerts anti-apoptotic and cytoprotective actions. It promotes the proliferation of dexamethasone-treated MC3T3-E1 cells, alleviates cytotoxicity and structural damage, and suppresses apoptosis by reducing caspase-9/3 activity and their cleavage products, decreasing the Bax/Bcl-2 ratio, and inhibiting mitochondrial cytochrome c release [22]. Moreover, chlorogenic acid activates HO-1, promotes Nrf2 nuclear translocation, and upregulates p-Akt, thereby suppressing superoxide production and alleviating oxidative stress through the PI3K/Akt-mediated Nrf2/HO-1 pathway [23]. Additionally, chlorogenic acid derivatives have been reported to enhance PGE2-stimulated bone matrix protein synthesis in osteoblasts via activation of p38 MAP kinase and SAPK/JNK pathways [24,25]. Furthermore, chlorogenic acid contributes to bone protection by modulating osteoclast activity. It inhibits RANKL-induced osteoclastogenesis while promoting osteoblast proliferation and differentiation, and its administration has been shown to counteract bone loss in ovariectomized rats [26]. Collectively, these multi-pathway regulatory effects position chlorogenic acid and its structural analogues as promising agents for accelerating bone repair [27], further substantiating the osteogenic potential of this class of compounds. The osteogenic effects of isochlorogenic acid B have been rarely reported, with only one study demonstrating its protective effect against hydroxyl radical •OH-induced damage in bone marrow mesenchymal stem cells (bmMSCs) [28]. Notably, a recent independent study employing SPR technology demonstrated that isochlorogenic acid B directly binds to TNF-α, blocking TNF-α/TNFR1 interaction and inhibiting NF-κB-mediated inflammatory responses, and exhibited significant anti-rheumatoid arthritis efficacy in the CIA rat model [29]. That study identified IB, along with daucosterol and vitamin E, as key TNF-α-targeting constituents of *P. forrestii*. The study not only validated the reliability of SPR technology in screening active constituents from *P. forrestii* but also suggested that IB plays a dual “anti-inflammatory and osteogenic” role within this medicinal plant. Combined with our finding that IB targets BMP2 and activates Smad signaling, these complementary studies strongly suggest that caffeoylquinic acid derivatives, particularly IB, exert their effects through simultaneous intervention in TNF-α-mediated inflammatory signaling and BMP2-mediated osteogenic signaling. This dual-targeting profile is particularly attractive for rheumatoid arthritis, where both inflammation-driven bone resorption and suppressed bone formation contribute to joint destruction. Collectively, these findings establish caffeoylquinic acid derivatives, especially IB, as a unique class of natural products that concurrently counteract inflammation and promote osteogenesis, constituting the primary pharmacodynamic material basis through which *P. forrestii* exerts its therapeutic effects on RA-related bone destruction.

Among the six BMP2-binding ligands identified, all share a caffeoylquinic acid backbone but differ in acyl substitution patterns and stereochemistry. Di-caffeoyl derivatives (IB, IC, and 3-O-caffeoyl-4-O-sinapoylquinic acid) generally exhibited more favorable binding energies than mono-caffeoyl ones, suggesting that two acyl groups enhance BMP2 engagement. Although IB and IC are constitutional isomers with identical molecular formulas, they differ in the stereochemical configuration of the quinic acid moiety (Figure 1C). This subtle configurational variation appears to have a profound impact on bioactivity, as IB consistently demonstrated superior osteogenic potency and a lower docking energy (−8.1 vs. −7.4 kcal/mol for IC) compared with IC. The more favorable stereochemistry of IB may allow the two caffeoyl groups to adopt a spatial arrangement better complementary to the BMP2 binding cleft, potentially contributing to its enhanced binding stability and downstream Smad activation. These observations offer a preliminary structural basis for prioritizing IB as the lead compound for further investigation. However, the osteogenic activity of IB observed in vitro warrants its further evaluation in vivo, and our forthcoming studies will focus on its efficacy in CIA and OVX models, particularly regarding its ability to restore bone formation under inflammatory conditions.

Despite these promising findings, several limitations should be acknowledged. First, the binding interactions identified by SPR-based fishing were not further characterized with kinetic parameters or affinity constants. Future work employing SPR kinetic analysis or microscale thermophoresis (MST) will be required to precisely quantify the binding affinities of the six compounds, to confirm whether IB indeed possesses the highest affinity among them, and to verify whether these ligands facilitate the interaction between BMP2 and its receptors at the molecular level. Such biophysical validation would also help establish target specificity, thereby strengthening the mechanistic evidence beyond the current SPR fishing and docking results. Second, although our data indicate that IB activates BMP2-Smad signaling, the detailed molecular events, including whether IB stabilizes BMP2 dimers, facilitates BMP2-BMPR interaction, or acts at a post-receptor level, have not been resolved in this study. The observation that IB failed to rescue LDN-193189-induced suppression suggests an action upstream of BMP receptor activation, but further mechanistic dissection is clearly needed. Third, we recognize that the use of MC3T3-E1 osteoblasts does not address the cartilage pathology of OA or RA. While this cell model is well-established and relevant for studying osteogenic differentiation and BMP signaling, it does not fully reflect the complexity of joint diseases. Future studies using co-culture systems or animal models will be necessary to evaluate the effects of IB on cartilage and overall joint pathology. Beyond these specific limitations, structure-activity relationship (SAR) studies and structural optimization based on the chemical scaffolds of active constituents such as IB are warranted, aiming to develop osteogenic drug candidates with enhanced potency and improved metabolic stability. The pivotal role of BMP2 in mediating the osteoprotective effects of IB should be further corroborated using BMP2 transgenic animals or conditional gene knockout models, which would provide definitive genetic evidence for target dependency in vivo. Additionally, approaches such as component knockout studies [30] could more definitively establish the causal relationship between specific compounds and the observed pharmacological activities.

## 5. Conclusions

In conclusion, this study identifies six caffeoylquinic acid derivatives from *P. forrestii* as direct BMP2-binding ligands, among which isochlorogenic acid B (IB) exhibits the strongest osteogenic activity. Mechanistically, IB activates BMP2-Smad signaling in a BMP-dependent manner, as evidenced by its failure to rescue LDN-193189-induced suppression. Importantly, IB reverses LPS-induced downregulation of the BMP2-Smad pathway and restores osteogenic gene expression under inflammatory conditions, highlighting its potential for treating inflammatory bone loss. In addition, IB is the first direct BMP2 ligand from *P. forrestii* with demonstrated activity in an inflammatory osteoblast model. Although in vivo validation is still needed, these findings provide a pharmacological foundation for developing caffeoylquinic acid derivatives as BMP2-targeting osteogenic agents.

## Data Availability

The original contributions presented in this study are included in the article/Supplementary Material. Further inquiries can be directed to the corresponding author.

## Supplementary Materials

The following supporting information can be downloaded at https://www.mdpi.com/article/10.3390/biology15161385/s1, Table S1: Mobile phase gradient. Table S2: Mass parameters (Sciex Triple TOF 4600 LC-MS). Table S3: List of primer sequences. Table S4: BMP2-ligands from *P. forrestii.* Figures S1–S6: MS^1^ and MS^2^ spectra of neochlorogenic acid (NCA), 3-*O*-caffeoyl-4-*O*-sinapoylquinic acid, chlorogenic acid (CA), cryptochlorogenic acid (CCA), isochlorogenic acid B (IB), and isochlorogenic acid C (IC). Figure S7: Safety evaluation of LDN-193189 in MC3T3-E1 Subclone 14 cells. Cell viability was assessed by CCK-8 assay following treatment with various concentrations of LDN-193189 (0, 0.5, 1, 2, 5, 10, and 20 µM) for 48 h. Figure S8: Representative Western blot analysis (corresponding to Figure 7B) showing the effect of isochlorogenic acid B (IB) on p-Smad1 protein expression in MC3T3-E1 Subclone 14 cells treated with LDN-193189. Figures S9–S12: Representative Western blot analysis (corresponding to Figure 10) showing the effects of IB on the expression of BMP2-Smad pathway-related proteins, including Smad1, p-Smad1, Smad4, Runx2, and β-actin, in LPS-induced MC3T3-E1 Subclone 14 cells.
