# Supplementary material for: BMP2-Binding Caffeoylquinic Acids from Periploca forrestii Promote Osteoblast Differentiation via Smad Signaling Activation
Source: Biology (Basel). 2026 Aug 13;15(16):1385. doi: 10.3390/biology15161385 (PMC13509640; doi:10.3390/biology15161385)
Supplement: Supplementary file 1 [file biology-15-01385-s001.zip › biology-4443548-supplementary.pdf]

Supplementary Information for

BMP2-Binding Caffeoylquinic Acids from *Periploca forrestii* Promote Osteoblast Differentiation via Smad Signaling Activation

Minghong Dong [1], Xinyue Wang [1], Xiongwei Liu [1,2], Tingting Feng [1,2], Chang Liu [1,2,*] and Ying Zhou [1,2,*]

[1] School of Pharmacy, Guizhou University of Traditional Chinese Medicine, Guiyang 550025, China; dmh99102@163.com (M.D.); wwwwxy996@163.com (X.W.); ashevy0819@163.com (X.L.); fengtingting040@gzy.edu.cn (T.F.)

[2] Guizhou Key Laboratory of Modern Traditional Chinese Medicine Creation, Guiyang 550025, China

* Correspondence: 19liuchang@163.com (C.L.); yingzhou71@126.com (Y.Z.)

Table S1. Mobile phase gradient

Time (min)	A%	B%	Time (min)	A%	B%
0–3	0–3	100–97	33–38	40–95	60–5
3–7	3–8	97–92	38–41	95	5
7–20	8–20	92–80	41–41.1	95–0	5–100
20–33	20–40	80–60	41.1–44	0	100

Table S2. Mass parameters (Sciex Triple TOF 4600 LC-MS)

MS parameter	value	MS/MS parameter	value
TOF mass range	50–1700	MS/MS mass range	50–1250
Ion Source Gas 1 (psi)	50	Declustering Potential (V)	100
Ion Source Gas 2 (psi)	50	Collision Energy (eV)	±40
Curtain Gas (psi)	35	Collision Energy Spread (eV)	20
Ion Spray Voltage Floating (V)	-4500/5000	Ion Release Delay (ms)	30
Ion Source Temperature (°C)	500	Ion Release Width (ms)	15
Declustering Potential (V)	100		
Collision Energy (eV)	10		

Table S3. List of primer sequences

Gene	Forward primer (5'->3')	Reverse primer (5'->3)
Bmp-2	TGGCTGGAGACTTCTTGAACTTG	AGAGAGGTGATCGGGCTGGA
Smad1	CTGTGGACGCTTTGGTGAAGAA	CGAGGAATGGTGACGCAGTTAC
Smad4	TGCTGGCATCGGTGTGGAT	GCTCGGTGAAGGTGAATCTCAAT
Smad5	AGCCGTGAAGCGATTGTTGG	CACACTTGCTTGGCTGTCCTG
Smad9	GGGAGAGGGAGGTTCAAGACAG	GGAGGCAGAGGCAAGCAGAT
Runx2	CCAGACCAGCAGCACTCCATA	CCGTCAGCGTCAACACCATC
Osterix	TCACCTGCCTGCTCTGTTCC	GCGGCTGATTGGCTTCTTCTT
COL1A	TGTCTGCCCAGCAAACAAAGG	TGGAGATGCCAGATGGTTAGGT
OCN	AGCAGGAGGGCAATAAGGTAGT	TAGATGCGTTTGTAGGCGGTCT
GAPDH	CGATGCCCCCATGTTTGTGA	GAGCCCTTCCACAATGCCAA

Table S4. BMP2-ligands from *P. forrestii.*

No.	Compound name	RT(min)	Adduction	Molecular Ion (*m/z*)	Error ppm	Formular
1	neochlorogenic acid	9.14	$[M-H]^-$	353.0879	-2.6	$C_{16}H_{18}O_9$
2	chlorogenic acid	11.57	$[M-H]^-$	353.0860	-1.4	$C_{16}H_{18}O_9$
3	cryptochlorogenic acid	12.41	$[M-H]^-$	353.0888	3.1	$C_{16}H_{18}O_9$
4	3-*O*-caffeoyl-4-*O*-sinapoy lquinic acid	17.13	$[M-H]^-$	559.1435	-4	$C_{27}H_{28}O_{13}$
5	isochlorogenic acid B	22.04	$[M-H]^-$	515.1198	1.2	$C_{25}H_{24}O_{12}$
6	isochlorogenic acid C	23.49	$[M-H]^-$	515.1211	1.6	$C_{25}H_{24}O_{12}$

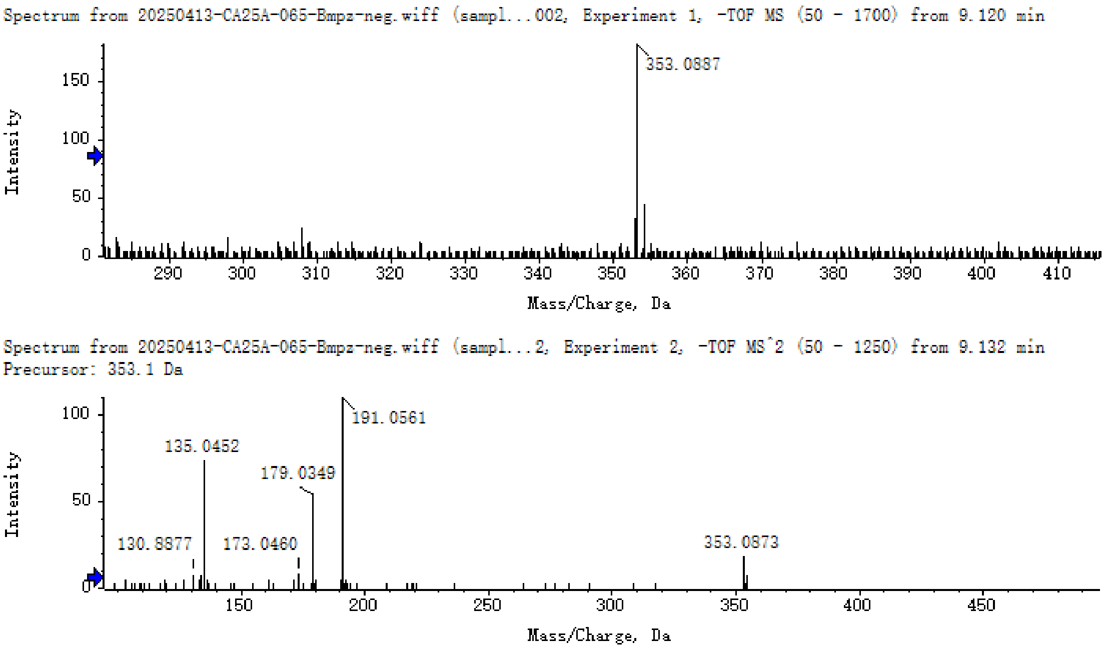

Figure S1. MS^1 and MS^2 spectra of neochlorogenic acid.

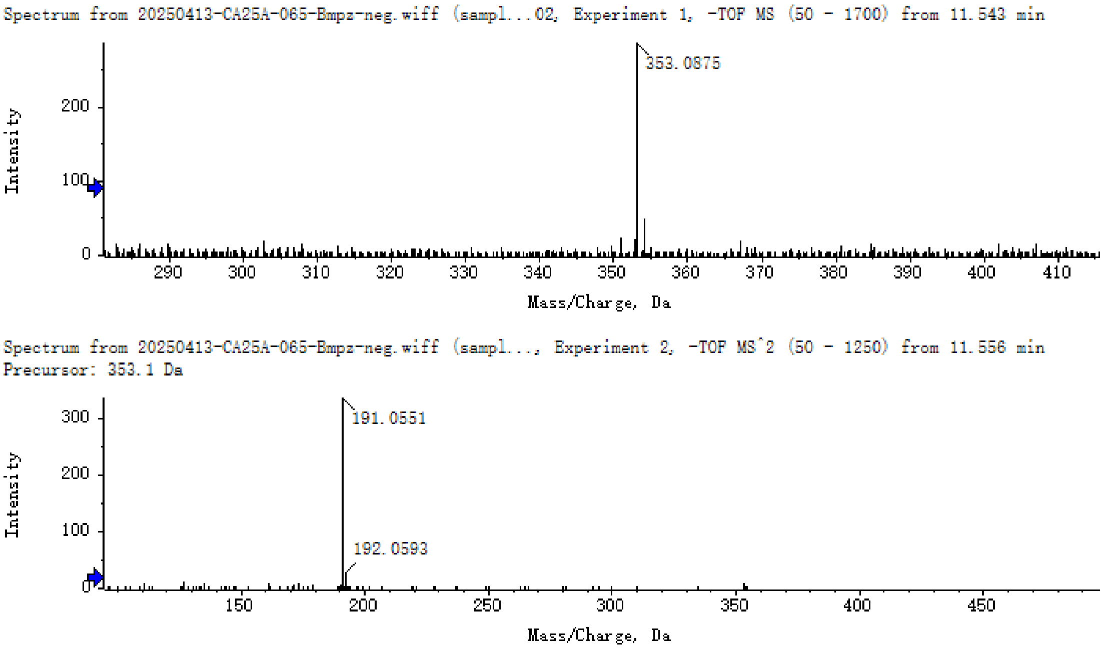

Figure S2. MS^1 and MS^2 spectra of chlorogenic acid.

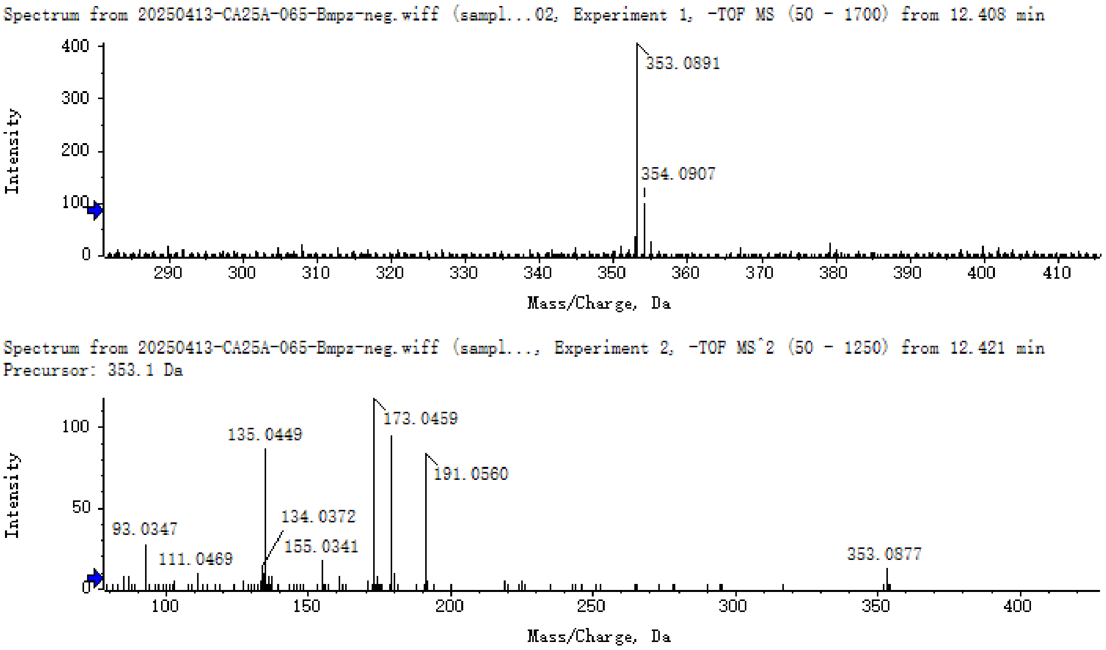

Figure S3. MS[1] and MS[2] spectra of cryptochlorogenic acid.

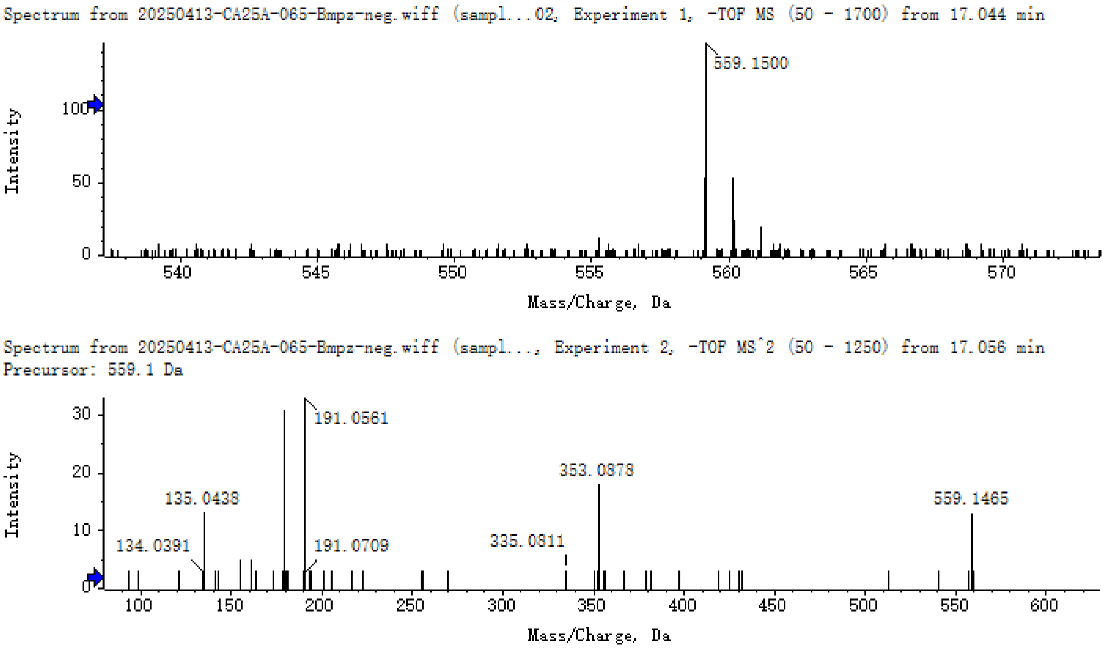

Figure S4. MS[1] and MS[2] spectra of 3-*O*-caffeoyl-4-*O*-sinapoylquinic acid.

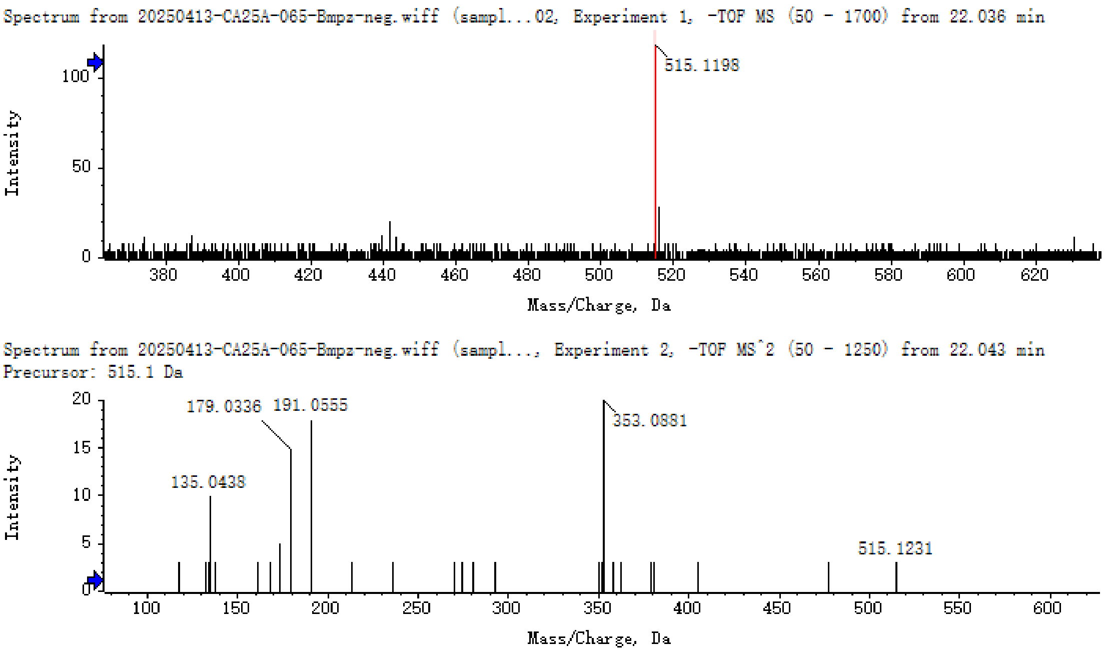

Figure S5. MS^1 and MS^2 spectra of isochlorogenic acid B.

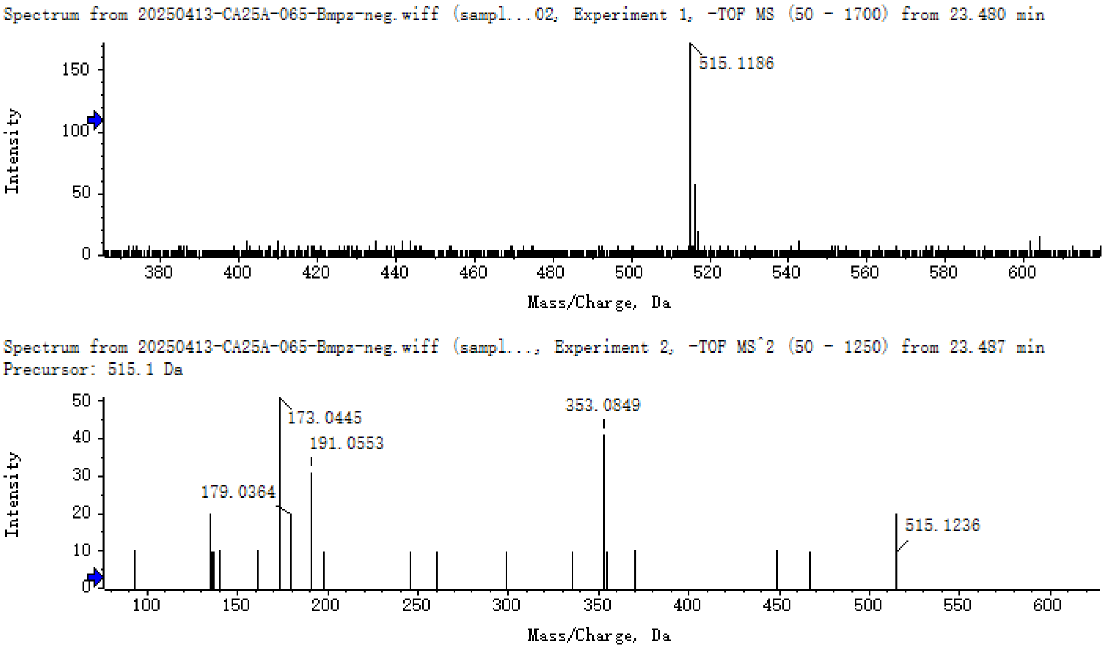

Figure S6. MS^1 and MS^2 spectra of isochlorogenic acid C.

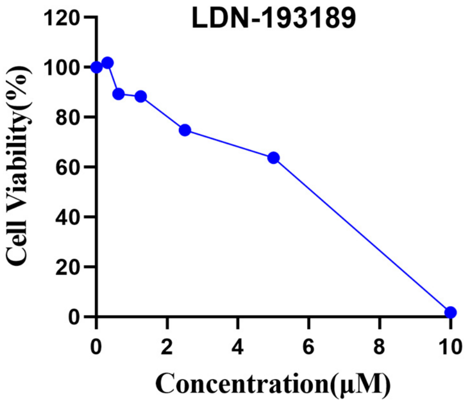

Figure S7. Safety evaluation of LDN-193189 in MC3T3-E1 Subclone 14 cells. Cell viability was assessed by CCK-8 assay following treatment with various concentrations of LDN-193189 (0, 0.5, 1, 2, 5, 10, and 20 μM) for 48 h.

Original western blot analysis of isochlorogenic acid B (IB) for three repeats

The effect of isochlorogenic acid B (IB) on p-Smad1 protein expression in MC3T3-E1 Subclone 14 cells treated with LDN-193189.

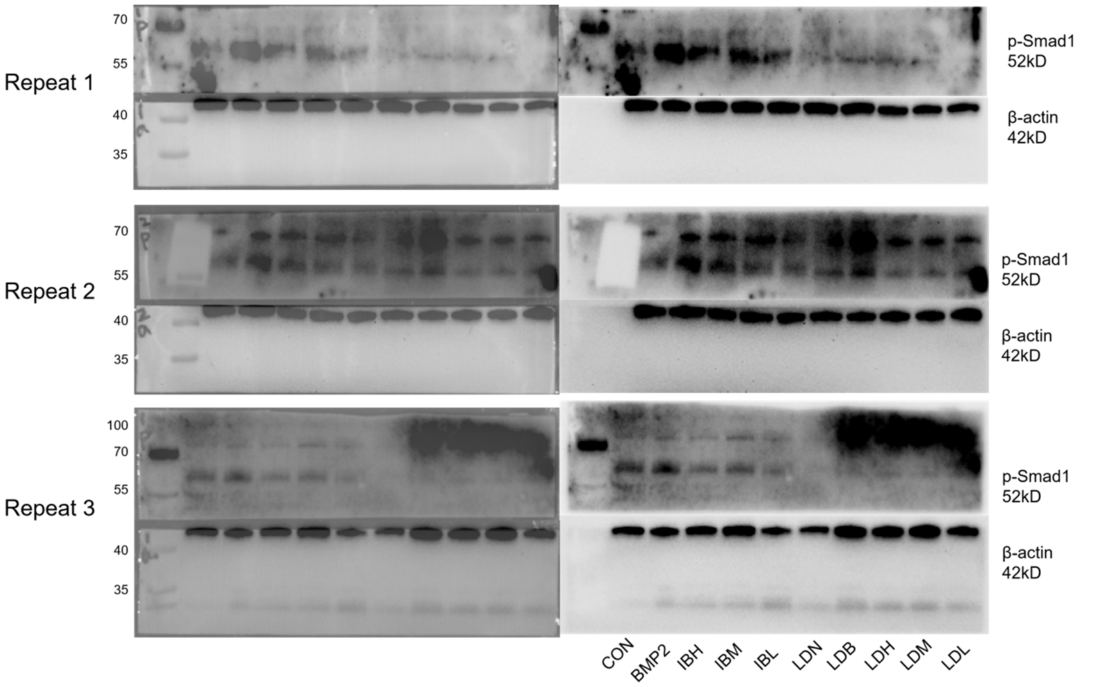

Figure S8. Representative western blot analysis (corresponding to Fig. 7B) showing the effect of isochlorogenic acid B (IB) on p-Smad1 protein expression in MC3T3-E1 Subclone 14 cells treated with LDN-193189.

The effects of IB on the expression of BMP2-Smad pathway-related proteins, including Smad1, p-Smad1, Smad4, Runx2, and β-actin, in LPS-stimulated MC3T3-E1 Subclone 14 cells.

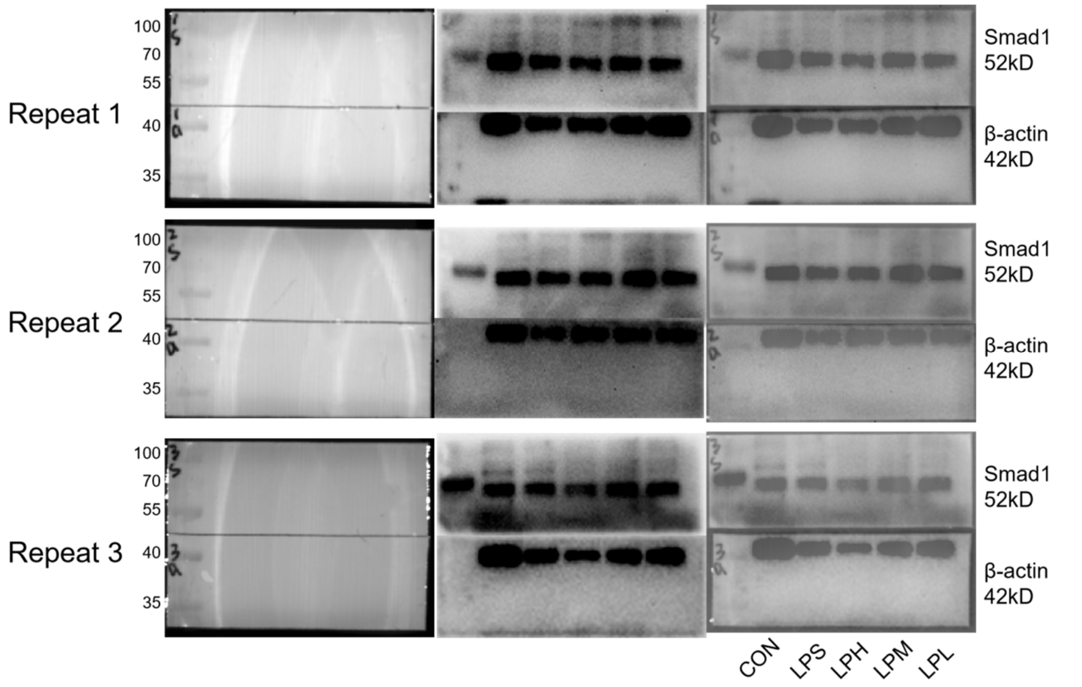

Figure S9. Representative western blot analysis (corresponding to Figure 10) showing the effect of IB on Smad1 protein expression in LPS-induced MC3T3-E1 Subclone 14 cells.

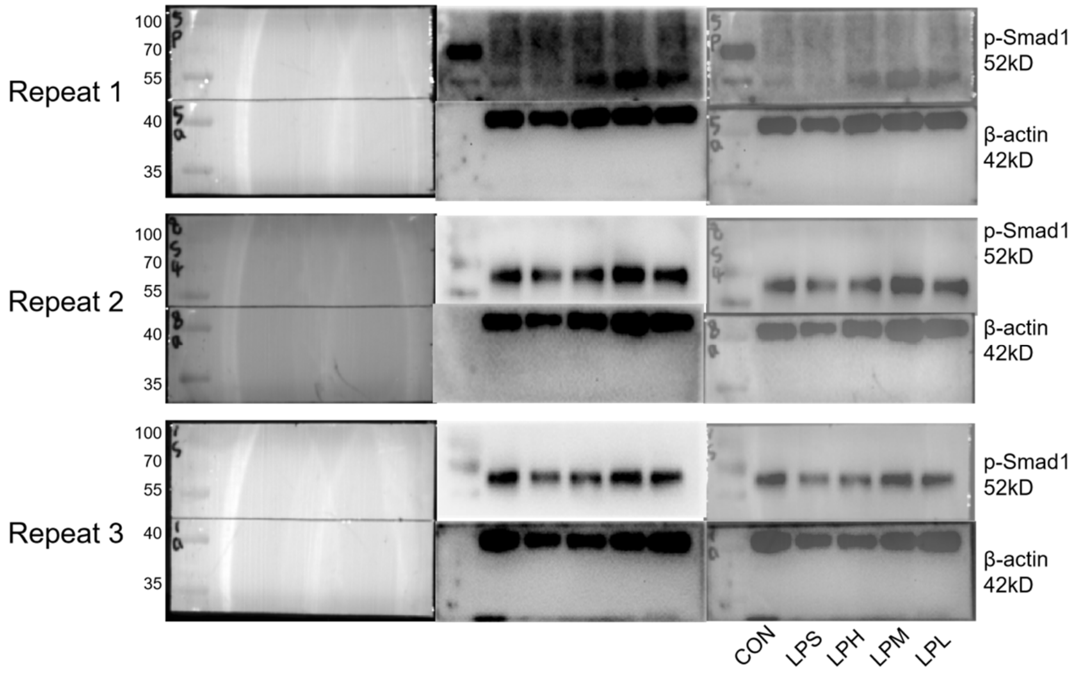

Figure S10. Representative western blot analysis (corresponding to Figure 10) showing the effect of IB on p-Smad1 protein expression in LPS-induced MC3T3-E1 Subclone 14 cells.

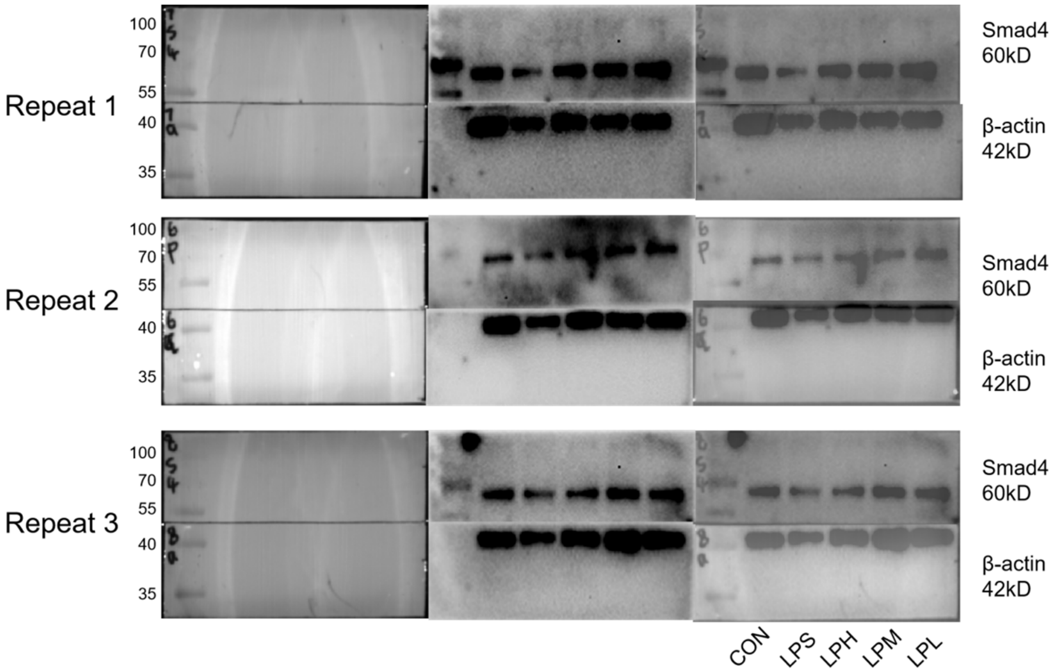

Figure S11. Representative western blot analysis (corresponding to Figure 10) showing the effect of IB on Smad4 protein expression in LPS-induced MC3T3-E1 Subclone 14 cells.

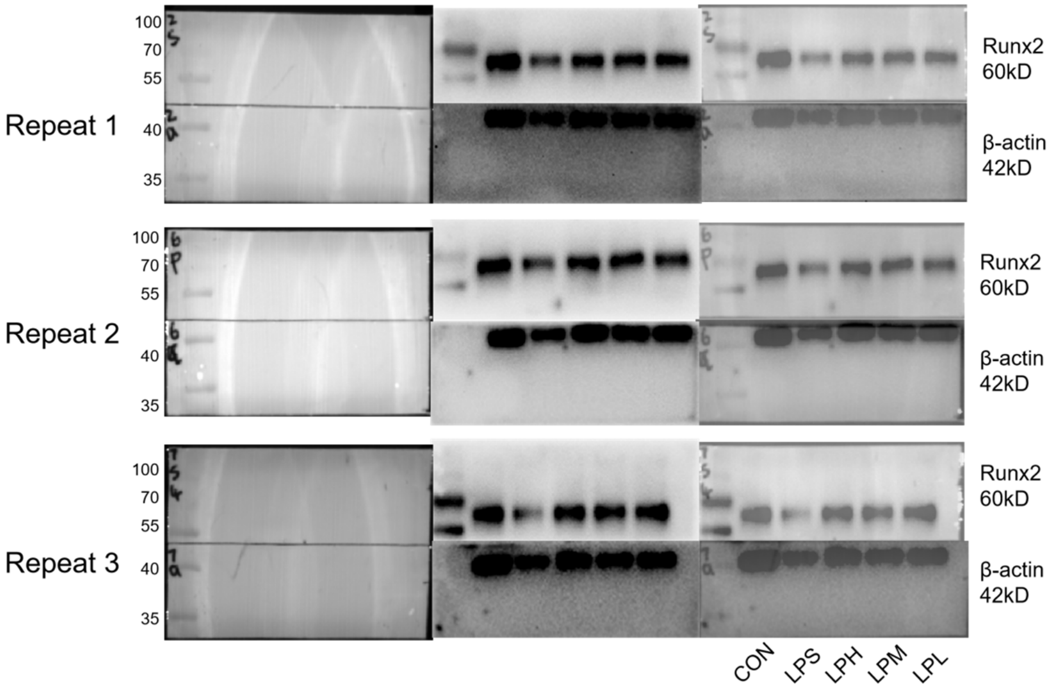

Figure S12. Representative western blot analysis (corresponding to Figure 10) showing the effect of IB on Runx2 protein expression in LPS-induced MC3T3-E1 Subclone 14 cells.
